# Yellow Field Pea Protein (*Pisum sativum* L.): Extraction Technologies, Functionalities, and Applications

**DOI:** 10.3390/foods12213978

**Published:** 2023-10-30

**Authors:** Nancy D. Asen, Rotimi E. Aluko, Alex Martynenko, Alphonsus Utioh, Pankaj Bhowmik

**Affiliations:** 1 Department of Food and Human Nutritional Sciences, University of Manitoba, Winnipeg, MB R3T 2N2, Canada; asennda@myumanitoba.ca (N.D.A.); rotimi.aluko@umanitoba.ca (R.E.A.); 2 Richardson Centre for Food Technology and Research, University of Manitoba, Winnipeg, MB R3T 2N2, Canada; 3 Department of Engineering, Dalhousie University, Agricultural Campus, P.O. Box 550, Truro, NS B2N 5E3, Canada; alex.martynenko@dal.ca; 4 ACU Food Technology Services Inc., 64 Laverendrye Crescent, Portage la Prairie, MB R1N 1B2, Canada; autioh@acufoodtech.ca; 5 Aquatic and Crop Resource Development, National Research Council Canada, 110 Gymnasium Place, Saskatoon, SK S7N 0W9, Canada

**Keywords:** plant-based protein, functionality, processing, green extraction

## Abstract

Yellow field peas (*Pisum sativum* L.) hold significant value for producers, researchers, and ingredient manufacturers due to their wealthy composition of protein, starch, and micronutrients. The protein quality in peas is influenced by both intrinsic factors like amino acid composition and spatial conformations and extrinsic factors including growth and processing conditions. The existing literature substantiates that the structural modulation and optimization of functional, organoleptic, and nutritional attributes of pea proteins can be obtained through a combination of chemical, physical, and enzymatic approaches, resulting in superior protein ingredients. This review underscores recent methodologies in pea protein extraction aimed at enhancing yield and functionality for diverse food systems and also delineates existing research gaps related to mitigating off-flavor issues in pea proteins. A comprehensive examination of conventional dry and wet methods is provided, in conjunction with environmentally friendly approaches like ultrafiltration and enzyme-assisted techniques. Additionally, the innovative application of hydrodynamic cavitation technology in protein extraction is explored, focusing on its prospective role in flavor amelioration. This overview offers a nuanced understanding of the advancements in pea protein extraction methods, catering to the interests of varied stakeholders in the field.

## 1. Introduction

Field peas are cool-season legume pulse crops globally grown for use in human and animal nutrition. In Canada and other countries, field peas are crops of economic interest and are used as export commodities. The most common field pea is the yellow cotyledon species, which is followed by the green cotyledon species and a few other species [1]. Pea seeds are high in starch, protein, and micronutrients such as vitamins and minerals but low in fats with varying proximate composition across different cultivars. 

While pulse crops are the most popular protein sources for human consumption in tropical and subtropical countries, their utilization is still in its infancy in Western nations [2,3]. Until more recently, soybean had been the most consumed plant protein, but there is a concern about allergenicity and genetic modification of the crop. Field peas have gained popularity due to their beneficial health effects, low production cost, and environmental sustainability [4,5]. The value of raw field peas is improved by processing into starch, protein, and fiber-rich fractions, and these ingredients are used in food formulations for nutritional enrichment or enhancement of techno-functional properties. Dry and wet fractionation or hybrid methods isolate pea protein constituents at different purity and functionality levels [6,7,8,9]. The outcoming products are subsequently used as ingredients in food formulations (i.e., stabilizers, emulsifiers, film-forming agents, and meat replacers). 

The nutritional value and functional properties of proteins are dependent on the quantity and quality of the protein. Pea proteins have a high potential to be utilized as ingredients in the food industry because of their relatively balanced amino acid profile when compared with other plant proteins like soybeans [10]. However, the use of pea protein as a food ingredient is impaired by limitations including the presence of antinutritional factors (impairs digestibility), objectionable flavor components, low net surface charge density, and a complex globular structure. Furthermore, extraction methods at high pH and temperature used in the preparation of commercial pea protein harm functionality and consequently lower performance in food applications when compared with the laboratory-prepared protein. High protein solubility is achieved under alkaline conditions; however, the probability of aggregation is also high, which leads to reduced protein solubility, especially at acidic pH conditions. A study by Malafronte et al. [11] showed that different protein morphologies are produced by varying the drying conditions. Shell formations of the protein could occur during spray drying, resulting in conformational buckling and rheological changes [11]. Several works have sought to valorize pea protein ingredients by the use of simple to cutting-edge technologies to improve functionalities and minimize adverse effects on the native protein conformation. This review will discuss the effect of different extraction and processing technologies on the functionalities of pea protein as well as its application in food formulations. We will review recent approaches in extraction methods to produce pea protein ingredients with higher yield, functionality, and applicability in food systems and identify technology gaps where information is needed to improve the flavor component. Furthermore, the functionality of pea protein will be compared with a standard plant protein ingredient like soybean. Statistics show five field pea market classifications in Canada; however, yellow field pea varieties account for >75% of the 3.8 million pea acreage [12,13]. Hence, a lot of our discussion will be around processing technologies for yellow field peas and some aspects of other varieties of field peas.

## 2. A Comparison of the Chemical Characteristics of Pea Protein and Soybean Protein

Yellow field peas, like other pulses, are a leguminous crop grown mainly for the consumption of dry seeds which are rich in macromolecules (protein, carbohydrates, dietary fiber, and resistant starch) and micromolecules (minerals, vitamins, and phytochemicals) but low in fats. The chemical composition of pea protein is affected by the cultivar (i.e., genotype), growth conditions, and the protein extraction methods used to produce concentrates and isolates. In addition, pea protein contains residual antinutritional substances (i.e., protease inhibitors) and natural pigments (i.e., tannins and anthocyanins). Maharjan et al. [14] analyzed the effect and interaction between genotype, rainfall, and temperature in different field pea genotypes grown in two different locations. The results showed variation in protein content across the different genotypes, and the effect on protein content came from the interaction between genotype and environment, but a significant difference was observed with the different growing environments. Phytic acid content was influenced by the growth environment as seeds grown in one location had 6.3–8.1 g/kg and those in another location had 4.8–7.5 g/kg. Phytic acid in the field of pea seeds affects the nutritional value by binding to essential minerals such as calcium, iron, magnesium, and zinc, causing reduced bioavailability of nutrients [14]. In this section, a detailed review of the chemical composition of pea protein will be carried out and compared with soybean, another major source of plant protein for the food industry. The effect of some processing methods on the chemical composition will be discussed.

### 2.1. Proximate Analysis

The chemical composition of yellow field pea flours as determined by Millar et al. [15] showed the following: protein content (21.0–22.0%), ash (2.76–3.5%), lipid (1.28–1.4%), moisture (10.6–13.35%), and total dietary fiber (14.0–15.0%). As a result of the low lipid content, the pea protein extraction process is relatively easy, faster, and cost-effective as the defatting process is not required [16]. However, Garcia Arteaga et al. [17] reported much higher values in the proximate composition of various pea protein species, as shown in Table 1. Dehulled field seed flours from 12 cultivars grown in different countries and harvest years showed variations in protein content (21.3–27.2%), ash content (2.5–3.6%), fat (1.9–2.5%), and starch (32.5–56.2%), and protein isolates from the same cultivars showed variations in protein (83.5–90.3%), ash (5.3–8.5%), and fat (4.7–9.0%) [17]. Lam et al. [18] investigated the physicochemical and functional properties of protein isolates derived from six pea cultivars grown in two different locations. The authors spotted differences in protein content (89.7–92.5%), ash (6.2–7.3%), and total lipids (2.3–3.5%) but stated that the differences had no practical significance. The total energy contributed by proteins for yellow field pea seeds is reported to be 24.4 and 26.3% [18], which satisfies the definition of high-protein foods [19]. Yellow field pea flour was also reported to provide at least 30% of the recommended dietary intake for zinc (3.78 mg/100 g), magnesium (114.2 mg/100 g), and potassium (1099.0 mg/100 g) [15]. Nikolopoulou et al. [20] showed that there was a significant impact on the proximate composition of field peas grown in three different locations for two years and established that high phytic acid content in pea seeds had a relationship with growing soils rich in phosphorus and low rainfall.

The crude protein content in a pea protein isolate obtained by alkaline extraction coupled with isoelectric pH precipitation (AE-IEP) was reported to be 83.33–84.67% (dry weight basis, dwb), but there were no significant differences between the protein content of isolates extracted at pH 8.5, 9.0, and 9.5 [21]. However, extraction at pH 9.0 produced isolates with low contents of lipoxygenase (beany flavor factor). Lipid content in this study was reported to be identical for all isolates (~1.47%), and the extraction method did not influence the ash and moisture contents. The composition of a commercial pea protein isolate prepared by AE-IEP and drum drying was reported to be as follows: total protein (68.85%), lipid (0.5%), total carbohydrate (26.6%), starch (0.31%), ash (3.53%), moisture (7.12), and legumin/vicilin (L/V) ratio (8.34) [22]. The protein content from the commercial isolate was low when compared to literature records of other wet-fractionated pea ingredients. Similarly, protein isolate compositions (dry weight basis, dwb) from different cultivars produced by isoelectric pH precipitation (IEP) after initial saline solubilization had protein content (81–89%), moisture (7.6–8.8%), ash (1.33–2.55%), lipids (0.5–5.5%), and carbohydrates (0.37–3.9%) [23]. 

As shown in Table 2, the major protein fractions in peas are the storage proteins, which are globulins (11S and 7S) and albumins (2S), with globulins making up the largest group of proteins (~65–85%). The electrophoretic profile of pea protein isolates under non-reducing conditions shows bands of approx. 10–105 kDa, which are assigned to dissociated hexameric legumin ~60 kDa subunits (α + β), intact vicilin fractions (α + β + γ) with bands of approx. 50 kDa, dissociated vicilin subunits (α + β) of approx. 30–37 kDa, and α, β, and γ subunits (approx. 14–20 kDa) [18]. The convicilin fraction is assigned the 70 kDa band, while lipoxygenase is assigned approx. 94–100 kDa. The same study reported 0.36–0.79 legumin/vicilin (L/V) ratios for the isolates and observed that the interaction between the cultivar and environment had no impact on these values. The polypeptide and allergen composition of pea protein could vary within the same cultivar grown under similar environment, harvest, and storage conditions. One reason is that the L/V ratio could change during the growth and maturity of the yellow field pea seeds. Dziuba et al. [24] carried out a proteomic analysis of a pea protein isolate using 2D electrophoresis and classified pea albumins as a heterogeneous group with 73 proteome accumulated spots in three molecular weight ranges of 50–110, 20–35, and 13–17 kDa over a broad isoelectric point range (pH 4.2–8.1). The pea albumin group comprises albumins (PA1), lectins, proteases, and protease inhibitors [24,25]. 

The colors of pea protein isolates differ and are dependent on the cultivar, as determined using the International Commission on Illumination (CIE) L*a*b* method. An analysis of 12 of the 2S proteins showed a range for L* (87–91), which signifies lightness; a* (−0.5–3.5) for the green cotyledon color; and b* (19–24), which is the yellow color. [17] In another study, pea protein isolate (PPI) was shown to be darker with L* (69.8), a* (2.21), and b* (19.25), when compared to the lighter soybean protein isolate (SPI) with L* (94.18), a* (0.09), and b* (−0.92); the color variations may occur due to presence of pigments in the seed flour or the protein drying method [35,36] The protein yield varies widely with cultivar and ranges from 34 to 62 g/kg, which is not dependent on the protein content of the isolate [17].

Soybean is an oil seed with approx. 20–30% lipid content depending on the cultivar [22]. The major proteins in soybean are glycinin (10.1S–14S) and β-conglycinin (7.1S–8.7S), making up >70% of the total protein [37]. A meta-analytical approach of data collected for 1944 samples of soybean meal obtained from 18 published papers (2002–2018) was used to quantify the chemical composition based on country of origin [38]. Similar to variations observed with peas, the results of the study showed that the country of origin affected the chemical composition and amino acid profile of the soybean products by great margins. The inconsistencies across the different locations arose from seed genotype, planting location, environmental growth and harvesting conditions, and storage and processing conditions. Analysis of the polypeptides showed that at ambient temperature and around neutral pH, the legumin-like protein is a hexamer with a molecular weight of 300–380 kDa and each subunit consists of a pair with an α or acidic unit (MW of ~35 kDa) and a β or basic unit (MW of ~20 kDa) linked together by a disulfide bond [39]. The glycinin hexamers are dissociable species that could fragment into smaller polypeptides and constituent molecules of trimers (3S–8S) under processing conditions like low ionic strength, heating, and pH. Glycinin can form a 7S trimer of ~180 kDa at pH 3.8 or low ionic strength (i.e., 0.1 M) and neutral pH [39]. Soybean has five classifications based on polymorphism of the glycinin fractions, but some compositional parameters differ within these groups, and since the proteins exhibit molecular heterogeneity, the groups differ in functional properties. The vicilin-like 7S soy protein consists of subunits α’ (57–72 kDa), α (57–68 kDa), and β (42–52 kDa); it exists as hexamers and trimers at low (<0.1M) and high (>0.5 M) concentrations, respectively, and is held together by non-covalent bonds [39]. The protein reversibly dissociates into 2S–6S subunits at low pH (<5) and ionic strength (<0.1 M). The content of 2S proteins in soybeans are very low and consist of protease inhibitors, cytochrome c, and α-conglycinin. All three subunits of β-conglycinin and α-conglycinin are recognized as potential food allergens in humans and different animal species [36,39]. 

### 2.2. Amino Acid Profile

As shown in Table 3, pea protein is limited in leucine as well as sulfur-containing amino acids (SCAAs) such as methionine and cysteine [17]. However, the leucine content of pea protein (5.7%) is slightly higher than that of soybean (5.0%), oat (3.8%), and hemp (2.6%) proteins [10]. Similarly, other studies reported higher lysine (4.7%) and phenylalanine (3.7%) for pea protein compared to soybean protein with values of 3.4% and 3.2%, respectively [10]. The essential amino acid composition of pea protein (23.6%) is slightly higher than that of wheat (18.9%) and soybean (19.9%) proteins and meets the WHO/FAO/UNU daily intake recommendation for adults [10]. Variations in amino acid composition are commonplace in pea protein, and responsible factors could be growth environment, germination, cultivar, storage, extraction methods, and processing conditions. Wet-fractionated pea protein isolate is reported to contain fewer SCAAs than dry-fractionated pea protein isolate because water-soluble albumins might be lost during acid precipitation and cysteine and serine residues are converted to dehydroalanine which is subsequently converted to lysine [40]. However, the amino acid and chemical score of wet-fractionated pea protein is superior to that of dry-fractionated pea protein with essential amino acids (EAAs) exceeding the FAO/WHO daily recommended level of 277 mg/g protein [40]. Furthermore, optimal unfolding and disruption of protein aggregates during wet fractionation facilitates increased protein solubilization and the higher presence of hydrophobic amino acids [40]. 

The different protein fractions (globulin, albumin, prolamin, and glutelin) vary in amino acid composition, and the major non-essential amino acids in globulins are asparagine, glutamine, glycine, arginine, isoleucine, leucine, phenylalanine, lysine, and threonine, while albumins are rich in tryptophan, lysine, and threonine [23,41]. Protein isolates obtained by lactic acid-assisted extraction exhibited improved amino acid composition because of the proteolytic activity of bacteria on the globulin and albumin fractions, which led to increased solubilization through the production of smaller polypeptides, peptides, and free amino acids [42]. Slight differences (~5%) were reported in the amino acid composition of pea protein isolates arising from different cultivars and extraction methods by Stone et al. [23], while Kaur Dhawali et al. [29] gave an update of up to 40% variations observed with some amino acids. Osen et al. [43] reported that the amino acid composition of pea protein isolates was not affected by the thermal and mechanical energy of 40–140 °C and 150 rpm, respectively, during extrusion, which suggests that there was no degradation of amino acids. The protein quality of soybeans is comparable to the quality of animal proteins because of the essential amino acid content. However, Gorissen et al. [10] showed that both pea and soybean proteins meet the WHO/FAO/UNU requirement at approx. 30 and 27%, respectively, and like most plant proteins, soybean protein is also limiting in SCAAs. 

### 2.3. Comparative Nutritional Aspects

The quality of any food protein is assessed by the amino acid composition and protein digestibility. The protein-digestibility-corrected amino acid score (PDCAAS) is an assessment model that has been in use for over 20 years, and it is based on the assumption that all amino acids have the same digestibility as crude protein and calculated using fecal digestibility. However, proteins are mostly digested in the small intestine, and an accurate way to determine amino acid release and availability is using ileal digestibility in a procedure called digestible indispensable amino acid score (DIAAS) [44]. The PDCAAS and DIAAS of soybean and pea proteins were determined in the ileum of growing rats, and the results showed no significant difference in both proteins with values of 98–99% and 94–97%, respectively [45]. Although SCAAs are limiting in both plant proteins, the authors showed relatively good availability and quality of methionine and cysteine in pea protein (92 and 98%, respectively) and soybean protein (89–91% and 94–97%, respectively) [45]. Another study determined the combined mean DIAAS and PDCAAS of soy products as 84.5 ± 11.4 and 85.6 ± 18.2%, respectively, using in vitro and in vivo assays [46]. Understandably, pea flour has lower protein quality than the extracted protein, and a study reported a low protein quality of pea flour (67.8%) when compared to cooked flour (69.19%) [47]. Commercial pea and soybean protein brands showed protein contents in the ranges of 77–81% and 61–91%, respectively [10]. 

### 2.4. Flavor Components 

Volatile (e.g., aldehydes, ketones, acids, pyrazines, and sulfur compounds) and non-volatile compounds (e.g., saponins, phenolic and alkaloid compounds) make up the flavor components of pulses [48,49]. Flavor is a combination of taste (i.e., non-volatiles perceived on the tongue), aroma (i.e., volatiles perceived nasally), texture (i.e., smoothness, viscosity, and sliminess), and trigeminal responses (i.e., brain in response to tactile or temperature stimuli). The off-flavor is a perception of an unpleasant taste or aroma and could be inherent in pea protein or develop during harvesting, processing, and storage due to lipoxygenase (LOX) activity, cultivar, harvest conditions, germination, and extraction methods [36,50,51]. An important inherent off-flavor in peas is the beany flavor which could be described as bitter, mouthcoating, rusty, nutty, metallic, or pea-like [52]. Although research shows it is difficult to attribute off-flavor to a single molecule, the presence of substances such as 3-methyl-1-butanol, 1-pentanol, 1-octen-3-ol, (*E,E*)-2,4-heptadienal, acetophenone, 1-octen-3-one, and 3-isopropyl-2-methoxypyrazine are reported to be responsible for off-flavor in peas [48]. The concentration of the substances in the pea ingredient contributes to the intensity or absence of the off-flavor, and only a few differences in flavor attributes were found among different cultivars [17,53]. Other compounds like hexanal can contribute to off-flavors but have no beany flavor in themselves [48]. The presence of beany flavor in pea ingredients is a challenge and limits their utilization in food applications. Similarly, the utilization of soybean products in the developed world has been limited by the presence of flavor compounds (i.e., ketones, aldehydes, furans, alcohols), and these compounds could interact with protein and turn on other flavor compounds [54,55]. Beany flavor in soybean is a result of the enzymatic oxidation of linoleic and linolenic acids catalyzed by lipoxygenase. Aromatic compounds linked with beany flavor are hexanal, hexanol, and *trans*,*trans*-2,4-nonadienal [54,55]. 

**Table 3:** Protein quality and digestibility of emulsion stabilized with pea and soy protein (g/100 g protein) and products (g/100 amino acids).

Essential Amino Acids	Pea Protein [56]	Soybean Protein [56]	DIAAS (Peas)	DIAAS (Soybeans)	FAO/WHO/UNU [57]
			Emulsions [58]	Milk [58]	
Threonine	3.80	3.90	3.86	3.73	2.30
Methionine	0.90	1.40	0.42	1.42	1.60
Phenylalanine	5.70	5.50	5.95	5.30	1.36
Histidine	2.40	2.50	5.60	7.10	1.50
Lysine	6.70	5.60	7.10	5.65	4.50
Valine	4.90	5.10	4.95	4.70	3.90
Isoleucine	4.40	4.90	4.85	4.74	3.00
Leucine	7.60	5.60	8.74	7.46	5.90
Tryptophan	0.90	1.30	3.23	2.82	0.60
Non-essential amino acids					
Serine	5.40	5.20			
Glycine	4.00	4.40			
Glutamic acid	16.40	20.50			
Aspartic acid	11.80	11.90			
Proline	4.40	4.90			
Cysteine	1.20	1.00			0.6
Alanine	0.71	4.20			
Tyrosine	4.00	3.90			
Arginine	7.80	8.40			

Several studies have been carried out to identify and reduce the odor-active volatile agents responsible for beany flavor in pulses. An article by Trindler et al. [48] gave a comprehensive review of the current state of knowledge on aromas and flavors associated with field pea protein. Off-flavors caused by inherent factors can not only be removed, masked, or modified but can also be prevented by breeding new cultivars. Off-flavors that develop because of external factors (i.e., storage temperature and moisture) can be controlled by careful handling of the peas and tuning of extraction methods. Physical, chemical, and enzymatic methods have been engaged in dealing with off-flavors in pulses. Thermal processing such as blanching at 60–100 °C can deactivate peroxidases and lipoxygenases [59,60]. Lactic acid fermentation of pea protein led to reduced or masked off-flavors by decreasing the content of n-hexanal (a lipoxygenase-derived molecule) and other contributors to the beany flavors [59,61,62,63]. Hexanal is a product of the degradation of unsaturated fatty acids and is the most abundant aldehyde detected in peas [64]. The content of aromatic compounds increased in fermented yellow pea flour, and the quantity of aromatic compounds produced was dependent on the bacteria strain and fermentation time [65]. Sensory analysis to evaluate flavor in pita bread and tortillas made from oven-roasted and micronized flour showed pitas from treated flour had higher aroma acceptability scores than those from untreated flours [66]. This result means that thermal treatment improves the flavor profile of the pea ingredient. Glycation of pea protein isolates with gum Arabic by incubation for 24 h improved the flavor profile remarkably (<1 ppm) through reductions in beany flavor markers [67]. Electronic tongue and sensory evaluation showed that enzymatic hydrolysis and the low-temperature Maillard reaction of pea protein reduced the bitter taste and increased the umami and salty tastes [68].

Recent advances have been made in monitoring flavor development, profiling, and reduction. Benavides-Paz et al. [69] monitored the development of volatile compounds during pH-optimized extraction of PPI using solvent-assistant flavor evaporation (SAFE), gas chromatography–mass spectrometry olfactometry (GC-MS-O), and gas chromatography–time-of-flight mass spectrometry (GC-TOF-MS). Wang et al. [49] performed aqueous solvent washing of air-classified pea-protein-enriched flour using different concentrations of ethanol and isopropanol to eliminate off-flavors. The effect of alcohol washing on volatiles, non-volatiles, proximate composition, and functionalities was compared between the untreated and treated samples. The volatile compounds were analyzed using headspace solid-phase microextraction (HS-SPME) coupled with gas chromatography/mass spectrometry (GC-MS). The results showed reductions in volatile compounds by 50 and 80% for ethanol and isopropanol washes, respectively. Some functionalities (i.e., protein content and in vitro protein digestibility) were enhanced by the alcohol washing while others (i.e., solubility and amino acid scores) were reduced after the washes. Another potential method for the elimination of flavors in plant protein sources was reported by Guldiken et al. [64], where different adsorbent resins, namely Amberlite-XAD16N, Amberlite-XAD7HP, Amberlite-XAD4, Sepabeads-SP207, and Diaion-HP20, were used to wash volatile and non-volatile compounds in lentil protein isolate. The results showed that treatments reduced the amounts of aldehydes, ketones, nitrogen compounds, alcohols, furans, terpenes, and enone in the protein, but total acids, aromatic compounds, and esters increased. However, the study concluded that the technique is a potential tool to be employed in the production of bland plant protein ingredients. 

## 3. Technologies for Pea Seed Isolation

**Figure 1:**
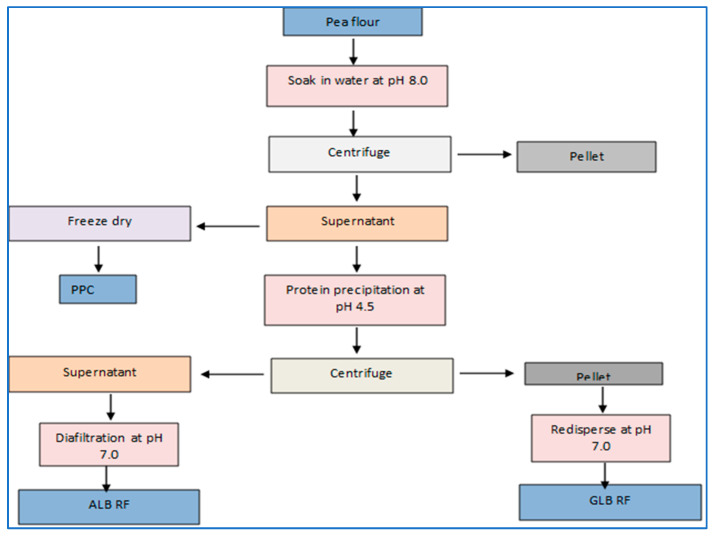
Flowchart showing a mild fractionation method adapted from Kornet et al [33]. ALB RF: albumin-rich fraction; GLB RF: globulin-rich fractions.

**Figure 2:**
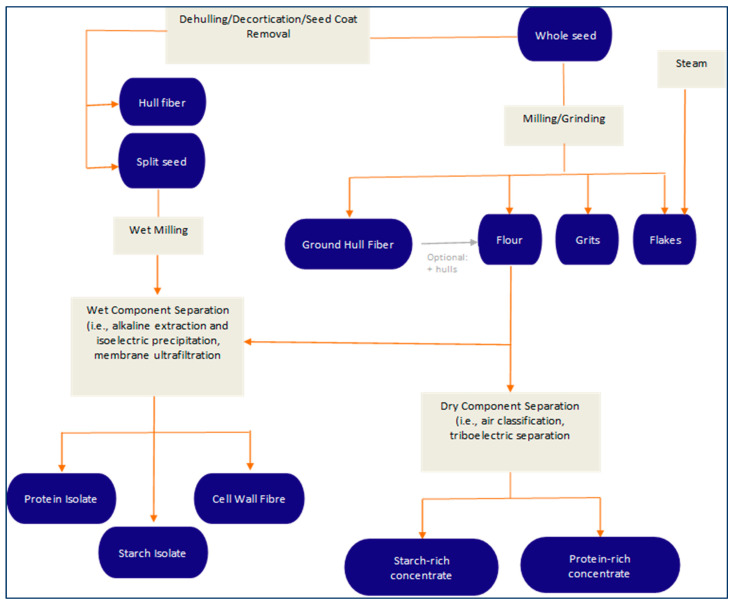
Wet and dry fractionation of pea protein. Adapted from Pulse Canada [80].

Field pea seeds contain 20–40% protein; 60% starch and dietary fibers; and other constituents, namely lipids (1.5–2%) minerals, vitamins, polyphenols, oxalates, saponins, and phytic acid [29]. Therefore, the separation of the protein fraction from starch and non-starch materials is necessary. Pea protein ingredients (flours, fiber, concentrates, and isolates) can be produced through dry, wet, or mild fractionation, and the different ingredients have varying protein contents (Table 4, Figure 1 and Figure 2). To maximize yield and enhance the nutritional, structural, and functional properties of the protein ingredient, it is essential to choose the appropriate methods matching the intent of the end-user. Before fractionation, the seeds are cleaned, dried, sorted, and then dehulled/split to preserve the functional properties of the derived protein ingredient [8]. Different fractionation techniques affect the protein ingredient differently; i.e., wet fractionation produces pulse protein with an essential amino acid content within the range of recommended daily consumption and enhanced emulsification and foaming properties, while dry fractionation preserves the native state of the protein and enhances hydration properties [70,71]. Higher protein purities are attained with wet fractionation, but the native structure and functional properties are altered to some degree. In this section, a few techniques in dry and wet fractionation will be discussed.

**Table 4:** Summary of processing and fractionation techniques.

Method	Plant Source	Objective	Summary of Finding	Author
Dry fractionation	Pea	Using dry milling in combination with air classification to improve protein enrichment	Approx. 50% purity and 77% protein yield were obtained using the method. The native functionality of the protein was preserved.	Pelgrom et al. [7]
	Peas, beans, chickpeas and lentils	Optimize milling using different settings to achieve maximum detachment of starch granules	Optimal detachment was achieved, but protein content was influenced by the intrinsic properties of the pulse.	Pelgrom et al. [8]
	Pea, lentils, and chickpeas	Air classification and electrostatic separation for protein enrichment	Higher protein purity (>60%), improved yield, less energy consumption, and preserved native protein functionality.	Xing et al. [9]
	Pea and faba beans	Effect of dehulling on physical, chemical, and technological properties of the fractions	Dehulling slightly increased the protein content of the fine fractions and improved starch enrichment of the coarse fractions. The techno-functional properties were not enhanced with dehulling.	Saldanha do Carmo et al. [72]
	Pea	Enhanced pea protein separation using Lorentz force-assisted charge carrier and triboelectric separation.	Protein content was increased by >100%.	Zhu et al. [71]
	Pea	Effect of the protein content of pea flour on physicochemical, antinutritional, and functional properties of air-classified protein fractions	Variations in protein content influenced the properties of air-classified pea flour.	Fenn et al. [73]
	Pea and chickpea	Determine the effect of relative humidity on particle dispersibility and flowability	Relative humidity above 70% affected the milling and air classification due to reduced particle dispersibility and flowability.	Politiek et al. [74]
	Mung bean, field pea, and cowpea	Compare the functional and rheological properties of dry-fractionated ingredients from mung bean, yellow pea, and cowpea	Protein content of the protein-rich fractions was dependent on the air classifier speed.	Schlangen et al. [75]
Wet and aqueous fractionation				
Aqueous/ultrafiltration	Pea	Mild wet fractionation using water only and continuous ultrafiltration	Method produced high-purity (75%) protein concentrates with improved solubility.	Möller et al. [76]
Alkaline extraction and isoelectric point precipitation	Pea	Compare protein functionality of isolates obtained from dry and wet (IP) fractionation	Wet fractionation produced isolates with high protein content, the presence of essential amino acids, and improved emulsification and foaming properties.	Zhu et al. [71]
	Chickpeas and green peas	Functional properties of protein isolates obtained by AE-IP method combined with modified salt dissolution precipitation	The purity of the globulin fractions was improved to >90%, and the protein composition played a major role in the functional properties.	Chang et al. [28]
	Pea	AE-IP extraction in conjunction with lactic acid fermentation	Protein content and yield were improved by 20–30%.	Emkani et al. [42]
	Pea	Compare the gelling properties of isolates obtained from different fractionation techniques	Gels from AE-IP in conjunction with ultrafiltration had good gel strength, but weak gels formed with AI alone.	Yang et al. [70]
	Pea	Mild wet fractionation coupled with isoelectric precipitation	Method produced both globulins and albumins; functionality was dependent on the dominant protein fraction in a sample.	Möller et al. [77]
Enzyme-assisted extraction method	Pea and flaxseed	Comparison of the properties of protein obtained from different extraction methods	Enzymatic solvent extraction produced high protein quality, and enzymatic extraction produced protein with good emulsifying properties.	Tirgar et al. [78]
	Pea	Investigate the effect of enzymatic hydrolysis on the techno-functional and sensory properties of pea protein isolates	The different proteases enhanced the properties of the protein and lowered bitterness.	Garcia-arteaga et al. [79]
Osborne fractionation	Commercial pea protein	Fractionation based on solubility in weak salt, water, alcohol, and weak acid or alkaline solution using Osborne fractionation with dialysis	Alkaline-soluble fractions (glutelins) were the most abundant (87.0%) while alcohol-soluble fraction (prolamins) was the lowest in both yield (1.52%) and protein content (57.7%). The other fractions had protein content >79.0%.	Adebiyi and Aluko [34]
	Pea flour	Fractionation of globulins and albumins using isoelectric point isolation	Albumins and globulins were isolated and showed good foam and emulsification properties, respectively.	Kornet et al. [33]

### 3.1. Dry Fractionation

The two major steps involved in dry fractionation are milling (pin, roller, hammer, or stone) and air classification (Table 4). Dehulling and dry milling are pre-processing techniques that optimize protein enrichment during air classification [8]. Dehulling of the seeds is carried out to remove the seed coat before milling into flour [81]. Dehulling could be dry or wet; dry dehulling involves pitting the seed surface by abrasion [81], microwave technology [82], and ultrasound treatment [83], while wet dehulling involves soaking or tempering seeds in water as well as chemical or enzymatic treatment [81]. Subsequently, the pea seeds are milled to particles with a diameter < 40 μm to detach the protein from other seed materials [8,9]. The particle size of the flour is important because too coarse or too fine milling could hinder the proper separation of proteins from the starch granules and other cellular materials. The particle size is dependent on the speed of the classifier wheel and retention times; for example, in the case of the milling of lupine seed flour, increased classifier speed reduced the particle size from 280 μm to 10–14 μm [8]. During milling, starch granules and cellular matrix rich in protein and fibers are released by grinding the cotyledon into powder [9]. This step is performed carefully to minimize the production of damaged starch. After milling, air classification is applied to separate the small protein bodies from the larger starch granules for protein enrichment, and the separation is based on the size, shape, and density of the particles [7,8]. Very fine milling impairs the efficiency of air classification because separation is easier when non-protein materials like starch granules and fibers have larger particle sizes than protein. Increased air classifier speed could produce protein-enriched fractions with higher protein content and enhanced gelation properties [75]. Combining air classification and electrostatic separation produced a pea protein product with higher purity (63–68%) than the 57% obtainable by air classification only [9]. An alternative method to air classification is the use of sieves, which is based on variations in particle size [81]. 

Several factors have been mentioned as affecting protein separation efficiency, namely seed hardness or softness and fiber, ash, and oil contents. High fat content in chickpeas (6%) was shown to increase the chances for flour particle agglomeration, which impaired separation, whereas low fat (1%) in lentils and peas promoted sufficient separation [9], suggesting that defatting of the flour before dry fractionation is one way to improve the protein content as the adhesive forces that impair flowability would be reduced [8]. De-agglomeration (DA) is a parameter that measures the flowability or dispersibility of the flours in the air during classification. The dispersibility of flours is influenced by particle–particle adhesion, high humidity, and size; i.e., finer flour particle sizes would disperse better under low pressure [8,74]. Dispersibility and air classification of oil-rich flours can be enhanced using food-grade flowability aids. Aerosil (12 nm) and potato starch (44 μm) were added to lupine flour (high oil content); the air classification was improved at low pressure, and increased protein content was observed [8]. 

Electrostatic separation has been used to improve the quality of protein ingredients produced by dry fractionation [8,9,73]. Different electrostatic methods have been adapted to combine with or replace air classification, and these include triboelectrification or tribo-charging, Lorentz force-assisted, electric and magnetic field separation [71]. The principle is the use of the triboelectric charging properties of the material to obtain protein concentrates. For example, proteins will carry higher charges on the ionizable R groups and the amino and carboxyl termini than carbohydrates. making separation easy. Combined electrostatic treatment and milling of lupine seeds produced a protein material with a purity 15% higher than that using air classification [84], and higher protein recovery was recorded in navy bean protein after two-stage triboelectric treatment [85]. Modest protein enrichment of fine-milled peas and lentils could also be achieved using electrostatic separation [9]. Dry pea protein with 72% purity was obtained by a hybrid method between air classification and electromagnetic processes [9]. Furthermore, the best selection of the charging wall tube material (aluminum, steel, nylon, and PTFE) and its effect on electrostatic selection in the protein enrichment of lupine were evaluated [86]. The finding was that the tube material did not affect the separation, but the hydrodynamic conditions of the process were important.

**Figure 3:**
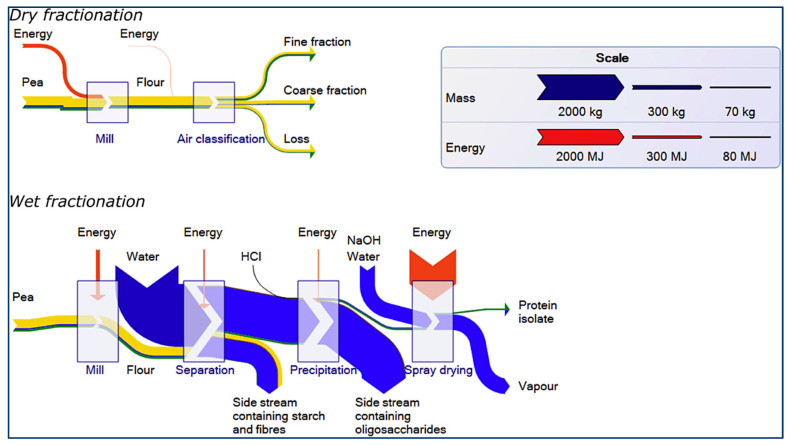
Energy consumption of dry and wet fractionation is shown as Sankey’s model. Adapted from Schutyser et al. [87].

The advantage of dry fractionation over wet fractionation is that the native functionality of the protein is retained, and dry fractionation is a more sustainable technique in terms of water and energy uses (Figure 3) [7,73]. For example, wet fractionation consumes >50 kg and 5.4 × 10^−5^ kJ/kg of water and energy for spray drying, respectively, per kg of recovered protein while dry fractionation has negligible water and energy (3.6 × 10^−7^ kJ/kg) usage [28]. During dry fractionation, more valuable components will be maintained in the protein matrix; however, antinutritional compounds such as protease inhibitors and lipoxygenases, which impair digestibility and contribute to the development of beany flavor, respectively, are also retained [87]. Overall, during dry fractionation, milder processing conditions (i.e., pH, temperature, and ionic strength) are required for fractionation [87].

### 3.2. Wet Fractionation

Wet fractionation is a conventional protein extraction method with the potential of producing high-purity (up to 95%) and high-yield (~60–90%) protein ingredients depending on the source [88] (Table 4). During dry milling, the small particles adhere to the larger ones after the structural break-up, which impairs the optimal separation into pure components [89]. However, the addition of water disentangles the particles to produce better separation [89]. However, this method has drawbacks such as the loss of the protein’s native state and functionality (e.g., solubility) resulting from the use of harsh processing conditions (salt, ionic strength, pH, and temperature) and the high cost of energy and water leading to an overall high production cost [90,91]. The final ingredient is dried into a fine powder (referred to as concentrate or isolate) by freeze-drying or spray-drying techniques for ease of storage and transportation. It is possible to strategically target steps in wet fractionation to optimize the production of protein ingredients with enhanced and varying functionalities [33].

#### 3.2.1. Alkaline Solubilization Coupled with Isoelectric pH Precipitation (AE-IP)

Alkaline solubilization coupled with isoelectric pH precipitation is a popular wet fractionation technique mainly because of the high-purity protein ingredients obtained (Table 4). This method is based on the solubilization of plant proteins, usually at pH 8–11, resulting from the increased electronegative charge on the protein surface; the solubilized proteins are then recovered by acid-induced precipitation at the isoelectric point, which is usually pH 4–6 for most pulses. At acidic pH values, the amide group of the protein gains an extra proton, which results in an electropositive charge, while the carboxyl group loses a proton at alkaline pH, producing an electronegative charge. To maximize protein yield and purity, processing conditions such as extraction pH, temperature, and flour–solvent ratio could be optimized [73,92,93]. Defatting is a pre-step in which the lipid content of the flour is reduced to improve hydrophilicity. Without defatting, protein–lipid interactions minimize solubility and impair protein yield during extraction [29]. During extraction, a mixture is prepared from pea flour by mixing with water and adjusted to alkaline pH; the mixture is then subjected to continuous stirring to dissolve the protein and other cellular constituents. The protein is then separated from the starch by passing the mixture through a centrifuge to obtain a protein-enriched supernatant. The supernatant is adjusted to the protein’s isoelectric point using hydrochloric acid (HCl), which causes protein precipitation that can be recovered as the solid portion after centrifugation. The precipitate is resuspended in water, neutralized with sodium hydroxide (NaOH), and frozen or spray-dried to obtain protein concentrates or isolates with purities of <90% or >90%, respectively, on a dry weight basis [94,95]. An alkaline pH environment during the extraction is achieved using the addition of KOH or NaOH, and because maximal solubilization is obtained, and a high protein yield is achieved [29]. Alkaline solubilization was shown to also cleave disulfide bonds, hence improving protein recovery and yield [96].

Conditions reported to affect AE-IEP protein extraction are the temperature, flour-to-solvent ratio, alkaline solution concentration, and processing time, and these conditions can be optimized to maximize protein yield and recovery [33,97]. The presence of compounds like phenolics, organic acids, lipids, and nucleic acids could cause protein degradation, which results in low protein yield and functionality [87]. The AE-IEP method reportedly depletes sulfur-containing amino acids (SCAAs) in the protein through the loss of albumins during solubilization at the isoelectric point, impairs the bioavailability of SCAAs (71%) and His (80%), and converts cysteine and serine residues in the protein to dehydroalanine, which could be transformed to lysine [98]. The electrophoretic profile of pea protein fractionated by wet methods showed weakly stained polypeptide bands around 11–30 kDa, which may be due to depleted albumins, and intense bands around 48–63 kDa indicating aggregate formation by the denatured proteins [98]. The secondary structure of wet-fractionated pea protein is also changed because hydrogen bonds are broken and electrostatic repulsion is induced during alkali treatment, which leads to the rearrangement of polypeptide chains to produce high contents of secondary structures like β-sheets and β-turns [98,99]. Similarly, the tertiary structure of pea protein is also altered by the action of the organic acids or alkali on the disulfide bonds that stabilize the internal structure leaving a loose spatial structure of the protein, which is reflected as exposed internal chromophores, i.e., Try, Tyr, and Phe [98]. The nitrogen solubility of dry-fractionated protein (i.e., 76 and 89% at acidic and alkaline pH, respectively) was found to be higher than that of wet-fractioned protein (i.e., 57 and 76% at acidic and alkaline pH, respectively), and this is directly linked to increased content of hydrophobic amino acids, increased surface hydrophobicity, and depleted content of water-soluble albumins [8,85,98]. Functional properties of different cultivars of spray-dried pea protein isolates extracted by AE-IP were determined by Cui et al. [51], and the result revealed that most of the functional properties were dependent on the cultivar. However, emulsion stability and foam properties (capacity and stability) were directly affected by the extraction method. In spite of the pitfalls linked with wet fractionation, modifications to this technique have been reported to produce protein isolates with preserved native structures, thereby maintaining the quality of functional properties. Chang et al. [28] used AE-IP extraction coupled with a modified salt dissolution precipitation method to extract legumin and vicilin fractions at a large scale from defatted green peas and chickpeas. The result showed that a high purity of the fractions was achieved (80 and 90% for legumin and vicilin, respectively). The result showed improved protein content of the pea globulin and fractions (~80–96%) and improvement in other functional properties (solubility, emulsion, and foam properties) when compared with the conventional AE-IP method [100].

Also, amino acid and chemical scores in wet-fractionated protein were reported to be higher than those in dry-fractionated pea protein [98]. This was evaluated by total amino acid content and total essential amino acid (EAA) content, which exceeded the FAO/WHO recommended level (277 mg/g), and the abundance of hydrophobic amino acids resulting from maximal structural deformation of the protein [98]. Lactic acid bacteria (LAB) were used to lower the pH during the AE/IEP extraction of pea protein [42]. The method resulted in a ~20–30% increase in the protein content and yield due to the increased solubility of the protein through the proteolytic activity of LAB.

#### 3.2.2. Ultrafiltration Processing (UF)

Ultrafiltration processing is a non-thermal, pressure-driven, and membrane-based separation technique with applications in protein fractionation, concentration, desalting, and clarification [96,101,102,103,104]. Ultrafiltration is a mild method because the native structure and functionalities of the protein are preserved, and it could be termed a green technique due to the absence of harmful chemicals and effluents [90]. Membrane UF technology is commonly characterized by a molecular weight cut-off (MWCO) and utilizes membranes with pore sizes of 0.001–0.1 µm, which act as physical sieves capable of retaining molecules with a molecular weight of ~30,000 kDa [90]. The MWCO is defined as the molecular weight above which ~90% of molecules are rejected by the membrane [91]. To obtain fractions with distinct sizes, the solubilized protein is sequentially passed through a smaller-sized membrane (e.g., 10 kDa), and the permeate is collected as the <10 kDa fraction. The retained solution is further passed through a bigger membrane size (e.g., 30 kDa), and the permeate is collected as the 10–30 kDa fraction while the retentate is the >30 kDa fraction. A reversed technique could start with a larger molecular membrane and the retentate collected from one size to the other. An addition to membrane UF technology is diafiltration, which involves the periodic addition of distilled water to the retentate during the process to reduce solution viscosity and increase the permeation rate through the membrane. UF technology has been widely used in dairy processing to improve the concentration of milk proteins or reduce lactose content in milk [92,93].

Membrane UF is used in combination with other techniques during protein extraction to produce ingredients with high native content and functionality. A comprehensive review of the application of the ultrafiltration technique in food applications was published by Ratnaningsih et al. [94]. An earlier study by Boye et al. [95] showed that the protein content of pea protein concentrates extracted by membrane UF and diafiltration increased by 4-fold compared to the flour content and was slightly higher than the protein content of the protein isolates obtained by AE-IP. More recently, Yang et al. [70] reported higher albumin content in pea protein fractions obtained through membrane UF and diafiltration than that obtained by AE-IP and the micellar precipitation technique. Also, the gel properties (capacity, morphology, and strength) and solubility of pea protein obtained from membrane UF of alkaline or salt extracts were superior to those of gels obtained from soybean protein [70]. Other studies reported the use of membrane UF and diafiltration in size-based separation and purification of pea protein and peptides from enzymatic digest. [96,102]. Hansen et al. [105] prepared salt-extracted PPI by coupling UF and diafiltration with mild solubilization of the protein at pH 7.5. The results revealed that protein content, yield, and functional properties of the laboratory-prepared PPI were enhanced when compared with a commercial brand and the method had the potential to be scaled up [105]. Additionally, Amat et al. [101] used UF technology to investigate interactions and complex formations between phytic acid, calcium, and pea protein fractions which could impair digestibility and bioavailability.

Limitations to UF technology are membrane fouling and concentration polarization, which decrease permeate flux [91]. Fouling reduces the efficiency of the process and reduces protein yield, and the remedy is the selection of appropriate membranes for protein separation [106]. Although the review shows that not much has been done with the UF technology in the processing of pea protein, it is clear from the few studies reported in the literature that UF is a non-invasive, easy-to-use, and green technology with proven results in protein processing.

#### 3.2.3. Micellar Precipitation

Micellar precipitation (MP) is a mild extraction method that produces proteins with a high native structure content. In this method, proteins are extracted in a salt solution at a neutral pH, and the insoluble materials are separated using centrifugation. Subsequently, the proteins are recovered through precipitation and the formation of micelles by the addition of cold water to the high-salt protein extract at different ratios. Micelles form in water as nanosized aggregates where the polar heads orient with the outer environment and the hydrophobic moieties are within the core. Another variation of this method is the reverse micellar precipitation which forms nanostructured aggregates of surfactant molecules in a non-polar environment containing water at the core of the structure. A thorough review of the process and applications of this method was carried out by Sánchez-Velázquez et al. [107] and Mondor and Hernandez-alvarez [108].

Although the MP technique has been reported as an efficient extraction method for proteins, the literature has scanty information about its use for pea protein extraction. A study by Yang et al. [70] showed that MP extraction favored the extraction of pea globulins as the albumins were lost in the supernatant. The same authors showed that MP-extracted PPI had high fluorescence intensity (unfolded), high protein content, and high surface charge when compared with PPI produced using other extraction methods. The high surface charge was attributed to the low albumin content of the MP isolates as albumins have higher isoelectric points. Also, the MP isolates formed gels with high mechanical strength (compressive stress = 80 kPa) comparable to that of gels from soybean protein isolate. Although the surface charge of the MP isolates was high, these isolates exhibited relatively low solubility (67%), which resulted from partial precipitation of proteins during centrifugation. However, an earlier study by Stone et al. [23] compared the functionalities of pea proteins from three cultivars extracted using AE-IP, MP, and salt extraction (SE). The result showed that the MP isolates for the three cultivars were low in solubility (43–49%), protein yield (31%), and surface hydrophobicity (14–16 arbitrary units). However, there was no statistically significant difference between the surface charge (−21 mV at pH 7) for all cultivars and extraction methods. Similarly, Tanger et al. [109] reported low protein yield with PPI extracted using the MP technique, which is due to high losses (28–40%) at the initial solubilization stage and others (23–36%) during the precipitation step when compared with AE-IP and SE. The authors further suggested that MP protein extracts had a high legumin/vicilin ratio and high native structure content compared to SE and AE-IP, which was attributed to the observed high denaturation temperature and enthalpy changes, respectively.

#### 3.2.4. Salt Extraction—Dialysis

The salt extraction method employs the basic salt-in (solubilization) and salt-out (concentration) principles of proteins [110]. The concentration step of salting out could be replaced with dialysis or membrane ultrafiltration. Yang et al. [70] compared the gelling properties of pea protein extracted using AE-IP with or without membrane UF, SE coupled with dialysis (SD) or membrane UF (SU), and MP. The results showed that protein contents of the SD and SU were not significantly different (~86%), but at pH 7, SD had higher surface hydrophobicity (732.19) than SU (594.91), which in turn had a higher surface charge (−23.47 mV). Consequently, SU had higher solubility (87%) at pH 7 than SD (63%) and the other extraction methods. Understandably, the isolates from SD and SU emitted higher fluorescence, which signifies a more compact conformation than the isolates prepared by AE-IP. SD produced particulate and weaker gels from fewer junction zones due to low legumin content while SU formed highly interconnected polymer-like gels facilitated by the presence of disulfide linkages. Another study reported that SE is the only method when compared with AE-IP and MP that retains both globulins and albumins after extraction as this method has no precipitation step [109]. The authors reported a relatively high yield (40%) but very low enthalpy change at pH 7 with (10%) or without salt, which signifies an unordered structure resulting from solubilization at pH 11.6. The optimal solubilization conditions suggested by these authors to maintain high native structure content with SE were pH 8 as reported by Stone et al. [23] or the use of a more neutral salt as reported by Sun and Arntfield [111].

#### 3.2.5. Water Extraction

Water extraction is a mild fractionation method that uses water with or without the adjustment of pH. This technology has the potential to produce proteins with high native structure content, but like all the methods discussed so far, there are limitations, which include low protein yield and recovery, low purity, and high water consumption. Geert et al. [112] showed that the use of less refined pea protein fractions may be an advantage because the presence of other compounds could enhance some functionalities like emulsion formation and stability, in addition to reduced consumption of water and energy as observed with other wet fractionation methods. Similarly, another study by Moller et al. [76] showed that multiple washing steps with water alone efficiently separate the proteins from the starch. Furthermore, the authors reported a higher protein purity of 75% after the ultrafiltration of the soluble proteins, and the recovered water was recirculated. This technique is not very popular but is worth studying because of the potential of sustainably producing protein ingredients with high functionality.

#### 3.2.6. Enzyme-Assisted Extraction (EAE) Method

Enzyme-assisted methods are green and environmentally friendly techniques [99,113]. The degrading enzymes act on major components such as cell walls (cellulose, pectin, and hemicellulose) to release protein bodies and break down proteins into smaller molecular sizes for improved solubility and ease of fractionation [114,115]. Protease activities will reduce the chances of protein denaturation and prevent the formation of complexes between the released proteins and other cellular components [115]. For example, proteases working under alkaline conditions have an optimum pH of 8–10 and a temperature of 45–60 °C [116]. EAE is shown to be a beneficial recovery technique for plant proteins and has more advantages than the conventional methods because the products formed have high purity and low production of toxic residues [117]. This method has been widely used in protein fractionation from different plant sources using either single or multiple enzymes. The drawbacks of this technology are that it is time-consuming and difficult to scale up and has high operational costs, high energy consumption, irreversible matrix alteration, and stringent protocols to maintain optimum conditions for the enzymes. However, scaling up enzymatic methods is very promising for the industry, and although it is difficult, it is also possible [118].

### 3.3. Scaling Up of Laboratory Extraction of Pea Protein Isolates to Industrial Scale

Different extraction and processing techniques have been used at the laboratory scale where process controls are relatively easy to manipulate. Scaling up these techniques requires high optimization of the processes at larger scales to maintain proper exposure to processing conditions and the purity, yield, and quality of the end product. Although scale-up is essential for industrial production, the information in the literature about pilot plants and industrial-scale pea protein extraction methods is very scanty. However, a few recent studies have reported some progress in the scaling up of pea protein extraction methods. Hansen et al. [105] determined the scalability of mild AE-IP or salt extraction coupled with ultrafiltration from the bench scale to the pilot plant scale and evaluated the effect of scaling on the functional properties of PPI. The authors reported some unavoidable differences in the extraction processes such as varying separation powers of the centrifuges and varying parameters of the ultrafiltration process. Furthermore, the process spanned two days, and precautions had to be taken to prevent microbial growth. Schmidt et al. [119] showed that upscaling of protein extraction from a laboratory centrifuge to a pilot plant decanter centrifuge was feasible, and the protein yield increased. Overall, the future of the innovations and methods employed in the extraction of plant protein depends on the ability of the researchers to scale up to optimized industrial-scale production levels.

## 4. Functional Properties of Pea Proteins 

The functional properties of pea proteins are the qualities exploited for food formulation and processing, and these properties are closely related to the physicochemical properties of the protein. To effectively use pea protein in food applications, some form of pretreatment is required to improve flexibility, surface properties, digestibility, and flavor attributes. These methods can be classified as chemical (e.g., glycation, conjugation, phosphorylation), physical (e.g., thermal, micro-fluidization, sonication, high-pressure homogenization, atmospheric cold plasma, hydrodynamic cavitation), and biological (e.g., enzymatic hydrolysis, fermentation, germination). In this section, the common functional properties of pea protein will be discussed along with conventional or cutting-edge technologies that have been employed in the modulation of the physicochemical properties and, consequentially, the functionalities. Also, we will discuss some novel processing methods already in use in soybean protein processing as potential techniques in pea protein processing.

### 4.1. Solubility 

Solubility is a measure of protein–solvent and protein–protein interactions and is largely dependent on a combination of factors such as the surface properties (charge and hydrophobicity) and non-covalent interactions of the protein. Karaca et al. [120] reported a correlation between the surface charge and solubility of native pea protein isolates; however, no relationship was established between the solubility and surface charge of commercial pea protein, probably due to the presence of aggregates [121]. Pea protein exhibits a pH-dependent solubility pattern, which is based on its amphiphilicity whereby solubility is higher below and above the isoelectric point [122]. This is because carboxylic groups of the protein are protonated at acidic pH and deprotonated at alkaline pH. At high pH, a negative surface charge on the protein facilitates the presence of electrostatic repulsive forces, and optimal unfolding of the structure is obtained [121]. Other functional properties of protein hinge on solubility for ease of homogeneity, flexibility, and mobility. Macej et al. [4] reported a direct relationship between the solubility of six pea genotypes and the emulsification activity index and indicated that the solubility of laboratory-prepared pea protein was better than that of commercial brands. This variance comes from the denaturation and formation of protein aggregates through some extraction processes and/or during heat-dependent spray drying [121]. The native structure of pea protein is globular and compact with less flexibility, and the net surface charge density is low as some ionizable (charged) groups are hidden in the core of the structure. High contents of the α-helical structure in proteins improve flexibility and solubility, as seen in animal proteins [123]. The secondary structure of pulse proteins consists largely of β-sheets, β-strands, and β-turns and has only a relatively small proportion of α-helical structures [124]. To improve solubility, the flexibility of the structure must be enhanced to expose the hidden groups [122]. Like most plant proteins, the solubility of native pea proteins at neutral pH is low (~20%) when compared with animal proteins, e.g., native β-lactoglobulin with >80% solubility at pH 7.0 [125].

Another factor that affects the solubility of pea protein is variations in the ratio of the different subunits. Going by Osborne’s classification, globulins are weak-salt-soluble, albumins are water-soluble, prolamins are alcohol-soluble, and glutelins are acid-soluble. Vicilin proteins are more soluble than legumins because they have a low molecular weight (LMW), are glycosylated, and contain no disulfide linkages (increased flexibility), while the α and β subunits of the legumin proteins are linked together by disulfide linkage, which contributes to structural rigidity [28,126]. Conversely, Liang and Tang [127] reported that legumin exhibits better solubility at pH 5 than vicilin. Laboratory-produced native and modified pea proteins have been shown to possess improved solubility of up to >80% (>pH 7.0). Another study showed that the L/V ratio in pea protein could affect solubility because vicilin is glycosylated, more hydrophilic, and contains higher amounts of charged amino acids like aspartic and glutamic acids [18].

A study was carried out to bridge the functionality gap between commercial and laboratory-prepared pea protein isolates by the treatment of the commercial protein with high-pressure homogenization (HPH) at 205 and 500 psi [121]. The results showed a significant increase in the solubility of the commercial pea protein powders after HPH treatment but impaired solubility for the laboratory equivalent, which had relatively high solubility before the treatment [121]. The solubility loss could be because of the exposure of hydrophobic groups, which may have induced protein–protein interactions. On the contrary, treatment of a commercial pea protein isolate (5%, *w*/*v*) with high pressure at 600 MPa for 5 min and heat treatment at 95 °C for 15 min was reported to impair its solubility [128]. Phosphorylation modification of PPI improved solubility by 171.21% as the hydration properties improved and the hydration layer was enlarged by the addition of polar phosphate groups. A combination of HPH and ultrasound-assisted Maillard reaction improved the solubility of pea protein by 80–98% at pH below and above the isoelectric point due to increased steric repulsion between protein molecules with attached carbohydrates on the surface [129]. A >50 kDa pea protein aggregate fraction obtained by heat treatment at pH 3 coupled with membrane ultrafiltration had better solubility at pH 3–9 than the native proteins [96]. This was because of enhanced protein and water interaction after the treatment when compared with the native protein. Another hybrid technique, which combined pH shifting (pH 7.0–12.0) with ultrasound and heating to modulate the structure of pea protein, led to an increase in solubility from 30% to 90% [130].

### 4.2. Water-Holding Capacity (WHC) and Oil-Holding Capacity (OHC) 

The WHC and OHC represent the total amounts of water and oil, respectively, that 1 g of a protein powder can absorb without expulsion. These properties are related to the texture, mouthfeel, and flavor retention of products and are based on the interactions of the protein with water or oil and other solutes [124,131]. WHC is a vital prerequisite functionality for the use of proteins in food applications such as meats and bread and may not have a direct relationship with solubility but with gelation [7,132,133]. This relationship was seen with an increase in WHC and gel strength when the oil weight fractions of the PPI-stabilized emulsion gels prepared at 37 °C for 6 h increased [134]. The WHC and OHC of pea proteins are reported to be comparable with those of soybean protein but superior to those of kidney bean protein, which makes PPI a suitable ingredient in the processing of products that require hydration and shortening [134]. 

Treatments to improve WHC and OHC of pea protein aim at structural modulation of the protein to expose the hydrophilic and hydrophobic groups, respectively. High energy media mill (HEMM)-treated pea dietary fiber (PDF) improved the WHC (37%) and OHC (123%) of a pea protein beverage because the treatment increased viscosity and steric properties [135]. High-intensity ultrasound treatment of pea protein powder (amplitude 0–100%) improved OHC (approx. 56%) as the intensity increased with a concomitant decrease in WHC (approx. 38%) [136]. Infrared heating (120 or 140 °C) of pea seeds and tempering to 20 or 30% moisture content before milling improved the WHC and OHC. Another study reported that ethanol washing of PPC improved the WHC by approx. 54% while the OHC decreased by approx. 34% as a result of reduced non-polar group content [137]. Furthermore, solid-phase and submerged fermentation of pea-protein-enriched flour using *Aspergillus oryzae, Rhizopus oryzae, Rhizopus oligosporus, Lactobacillus plantarum,* and *Bacillus subtilis strains* significantly improved both WHC and OHC due to the optimal exposure of polar and non-polar groups [138]. Additionally, pea protein blended with other flours was shown to improve WHC or OHC. Another blend between PPI and brown rice protein isolate (ratio 4:6) crosslinked with microbial glutaminase (1 U/g) improved the WHC by 91.6% while the OHC was reduced by approx. 39.3% [139]. This is because crosslinking at optimal substrate concentration produced a continuous network structure that could trap water [139]. However, enzymatic treatment and crosslinking of blended pea protein (with hemp 1:1, rice 3:2, and oat protein 1:1) reduced WHC, and increased OHC was observed in a few combinations [140]. Another study incorporated oat β-glucan as a fat substitute in a 1% pea protein yogurt and improved the WHC by 6% as a result of the dense network structures formed by the polysaccharide [141].

### 4.3. Foaming Capacity and Stability 

Foams are two-phase dispersion systems of air cells separated by a thin continuous liquid layer, the lamella [142]. Foaming capacity is the ability of a substance under certain conditions (pH, ionic strength, and temperature) to quickly form a film around air bubbles in a food system (e.g., whipped cream, ice cream, and meringue), and foam stability could be evaluated as the volume of foam and liquid drainage that occurs over a fixed period [23,143]. The interest in foam properties is driven by the sensory pleasure derived from foamed products (e.g., the feel of ice cream or meringue kisses), and the sensory property of the product is dependent on the size distribution of the air bubbles within the food system. Foam systems with smaller and evenly distributed air bubbles produce food products with more appealing sensory properties. The capability of a protein to facilitate foaming is directly dependent on performance at the water/air interface [144]. Factors that influence foam properties include flexibility, film formation, dispersibility, and solubility [145,146,147]. Pea albumin 1 (PA1) has a foaming ability superior to that of pea protein concentrate (PPC) and globulins because of the ability to form air bubbles at least four times smaller than globulins, which was observed as high foam overrun (258%) and stability after 272 min [33]. Lower foam overruns (<81%) and stability (<70 min) were produced by the PPC and the globulin fractions due to larger size and higher surface charge when compared to the PA1 [33]. The results may be attributed to the higher surface activity of PA1 in the first 10 s, which facilitates the formation of a stiffer interfacial layer, in addition to the formation and retention/stability of higher foam levels than the PPC and globulin fraction [33].

The processing environment and pretreatment techniques influence the foam properties of proteins. For example, treatment of PPI at 90–100 °C and pH 5.0 (isoelectric point) significantly reduced the foaming ability as both conditions reduced surface properties and increased electrostatic attractive forces [148]. The unfolded structure of proteins could optimize foam properties because of the exposure of hydrophobic groups to the surface. The use of high-pressure supercritical CO2 treatment improved foam stability by unfolding the structure and creating affinity between CO2 and the hydrophobic moieties to improve the surface properties [149]. However, Lam et al. [18] showed that foaming capacity is not dependent on the intrinsic properties of the protein (e.g., surface properties and L/V ratio) but on structure and conformation (e.g., flexibility), which allow for quick adsorption at the lamella. Variations in foam properties were observed for protein isolates derived from different cultivars and extraction methods by Stone et al. [23] with results that showed better foaming capacity for salt extraction (SE) isolates and better foam stability for AE-IEP isolates. This observation may be because SE isolates were able to unfold, quickly adsorb to the air/water interface, and reduce the surface tension while AE-IEP proteins produced stable foams indicating strong interfacial films were formed by the adsorbed proteins. Similarly, PPC from different cultivars obtained by air classification had a wide range of foaming capacity (208–455%), which was positively correlated with the protein content [73]. 

Chang et al. [150] treated pea vicilin by pH shifting, controlled heating, and high-intensity ultrasound or a combination of methods to determine their effects on functional properties. The results showed that pH shifting and controlled heating at 80 °C for 30 min improved the foam capacity by approx. 105% when compared to the untreated pea vicilin (73.53%) while foam stability was not different from the control. During controlled heating, soluble aggregates could be formed, β-sheets were converted to α-helices, and the surface properties (i.e., surface hydrophobicity) were enhanced [150]. Similarly, Asen and Aluko [96] reported improved foam capacity and stability (>10 and >7%, respectively) for soluble pea protein aggregates prepared at a controlled temperature (100 °C for 30 min) and at different pH values coupled with membrane UF (>50 kDa MWCO), especially at pH 3.0, 7.0, and 9.0. Another study showed that the addition of tea saponin to PPI (50 mg/mL) improved foam capacity (210%) when compared with PPI alone (113%), while stability was improved at a lower protein concentration (10 mg/mL) [151]. A high protein concentration would facilitate optimal adsorption at the air–water interface to form a thicker and larger surface area, but foam stability was favored by a low protein concentration due to cell coarsening and coalescence [151]. The combined surface activity of 0.4% saponin–PPI complexes at the air–water interface enhanced foam capacity (263.33%) [151]. Also, Shen et al. [152] showed that the addition of TWEEN 20 to PPI improved foam capacity via protein displacement by the more effective nonionic surfactant at the interface, and foam stability was achieved by network formation on the protein film.

### 4.4. Emulsification Properties 

Emulsification properties are the most widely studied functional properties of pea proteins, and the reason is that oil-in-water (O/W) emulsions are common in several food applications (e.g., milk, yogurt, soups). Emulsifiers are required to reduce the surface tension at the oil–water interface to stabilize the emulsions, and pea proteins have been identified as potential natural emulsifiers because of their physicochemical properties. Research has shown that commercial pea proteins have limited use in emulsification as functionality is reduced due to greater denaturation and protein aggregation resulting from harsh processing conditions (e.g., AE-IEP, hot air oven, and spray drying); hence, pretreatment is required [121,153]. Pretreatment of native proteins is required because of the compact and globular conformation that prevents the optimal encapsulation of oil droplets. In previous reports, cultivar and extraction methods have been reported to play a role in determining the emulsification functions of proteins [154]. Intrinsic (molar mass, hydrophobicity/hydrophilicity ratio, charge, and conformational stability) and extrinsic factors (pH, ionic strength, and temperature) play an influential role in the determination of the emulsifying properties of proteins [155]. Unlike foam properties, the emulsification properties of proteins have been shown to be favored by the surface charge because flocculation can occur when the net charge around the oil droplets and the electrostatic repulsive force are reduced [16]. Monomodal distribution and small oil droplet sizes are some indices of a good emulsifier, and the stability of an emulsion is achieved by the formation of a thick viscoelastic film at the interface and the presence of steric hindrance and electrostatic interactions [156]. The larger size and higher net charge of PPC and the globulin fraction facilitated the formation of stable emulsion droplets, while albumin-stabilized emulsions were only stable at higher protein concentrations [33].

The literature is replete with studies on different pretreatment methods used to modulate the emulsification properties of pea protein. Just to mention a few, a study showed that grinding PPI to powder for 10–20 min significantly reduced the particle size of the droplets, increased the surface charge, and improved emulsion stability [157]. Combining pH shifting with ultrasound and heating led to improvements in the solubility and surface hydrophobicity of PPI by 33 and 30%, respectively, and a subsequent improvement in emulsion stability [130]. Heat treatment of PPI at 95 °C for 30 min also improved emulsification properties compared to those of the unheated pea protein, and higher proportions of vicilin and the basic subunit of legumin became adsorbed to the oil–water interfacial layer of the emulsions [158]. In addition, ultrasonic drying at 30 °C produced PPI with smaller protein aggregates and enhanced solubility and emulsification properties compared to those of the continuous sheet-like morphology formed by conventional hot air drying at 60 °C [153]. High-intensity ultrasound treatment of water-soluble pea protein fractions at 200, 300, and 500 W for 5, 10, and 20 min changed the secondary and tertiary structures and improved solubility and foam stability but impaired the emulsification properties [159]. The interfacial and emulsification properties of pea proteins were enhanced by high-intensity ultrasound treatment (57–60 W.cm−2 for 5 min) at 50% amplitude [160]. PPI–κ-carrageenan-complex-stabilized emulsions exhibited enhanced emulsion activity and stability, which was influenced by the hydrophilic groups from the κ-carrageenan [161]. Likewise, emulsion activity indices of PPC and PPI extracted from roasted pea seed (150 °C for 10–20 min) were enhanced at pH 7.0 due to improved solubility [162].

### 4.5. Gelation Properties 

Gels are formed when large molecules crosslink to form a 3D structure that is an intermediate between a solid and a liquid; these structural changes can be induced by heat, chemical, and enzymatic treatments. The ability of proteins to form gels is evaluated as the least gelation concentration (LGC), which is the lowest protein concentration required to form a self-supporting gel; the lower the LGC, the better the gelation ability of the protein. During gelation, various steps occur, including denaturation, aggregation, and formation of a protein network. Functional groups such as the sulfhydryl are exposed when the structure is unfolded, and irreversible aggregates are formed through disulfide bridge, hydrogen bond, hydrophobic, and/or van der Waals interactions [132]. The formed gels become self-supporting at a sufficient protein concentration, which is dependent on the protein source. Protein gels modify the texture of foods (e.g., seafood and meat replacements), and pea protein gels have attracted attention as an alternative to soybean protein, but the weaker gelling properties of soybean proteins have limited applications in food formulations [163,164]. 

Gelation is influenced by factors that include the extraction method; relative ratio of protein subunits; solubility; protein content; and other cellular materials like carbohydrates, lipids, and fiber [34,70,165]. The vicilin fractions of pea protein have been shown to possess better gelation ability than the legumin fractions, and PPI with a higher vicilin proportion formed better gels [164,166]. Legumin subunits under heat treatment (90 °C) will denature and form large insoluble aggregates through disulfide bonds, while vicilin forms smaller aggregates stabilized by non-covalent interactions [70]. Pea protein gels could be described as firm and flexible for meat analogs or weak gels for semi-solid foods like tofu and yogurt [70,164], and the optimal pH for gelation of pea protein has been reported to be around neutral [34,120,166]. Commercial PPI has been reported to produce weak gels and higher (20–23%) LGC [34,164]. However, various types of processing could improve the quality of gels prepared from pea proteins. Transparent and thermo-reversible pea protein gels like gelatin were prepared by the ammonium sulfate precipitation method at pH 2.4–4.2, 10–15% protein concentration, and a compressive stress of ~6.32 kPa, which formed gels dominated by hydrogen bonds but no disulfide bonds and hydrophobic interactions [98]. The rheological and structural properties of heat-induced pea protein gels were enhanced by pH-shifting treatment where the protein solution was prepared in a buffer at pH 7.4, adjusted to pH 12, and then reverted to pH 7 followed by heating at 92 °C for 1–2 h to form gels [98]. A uniform polymer-like gel network microstructure was obtained by this method because the treatment enabled the optimal unfolding of the protein structure and exposure of reactive groups to facilitate intermolecular interactions [98].

An earlier work by Sun and Arntfield [111] reported better gel quality and lower LGC of native PPI (14.5%) after the addition of 0.3 M NaCl, which reduced electrostatic repulsive forces and increased intermolecular interactions between the protein molecules with subsequent formation of a network. Enzymatic treatment (microbial transglutaminase) of gels containing 20–23% pea protein improved the structural quality, producing firmer and more flexible gels suitable for formulating meats and seafood [164]. A study of the effects of different extraction methods on pea protein gel properties showed that PPI extracted by micellar precipitation or UF of alkaline- or salt-extracted isolates formed gels with good compressive strength (60–80 kPa) because of optimal unfolding and the formation of strong protein–protein interactions [70]. The physical properties of meat analogs prepared by high-moisture extrusion (50 g/100g) of pea protein were enhanced by the addition of *Haematococcus pluvialis* residue (HPR) at 10–40 g/100 g; HPR gave the extrudate a reddish meat color and a loosened layered fibrous structure [167]. Another study improved the properties of pea protein aggregates prepared at ≥90 °C by the addition of κ-carrageenan (0.5%) and a low protein concentration (7.5%) [163]. The surface hydrophobicity of the aggregates was modulated, which increased the capacity of the proteins to act as building blocks to form a three-dimensional network and subsequently produce gels with superior mechanical strength, while the untreated protein could not form gels [163]. Additionally, treatment of PPI with novel cold atmospheric plasma sources produced soluble protein aggregates through disulfide linkages and increased the surface hydrophobicity and β-sheets, which resulted in the formation of a strong 3D gel network [168]. 

### 4.6. Digestibility of Pea Protein 

The nutritional quality of a protein is defined by the FAO/WHO based on the amino acid content and in vivo bioassay digestibility (FAO/WHO/UNU 2007). The FAO/WHO proposed the description of the nutritional quality of protein using digestibility based on individual amino acids (digestible indispensable amino acid score, DIAAS) rather than digestion based on the whole amino acid composition (protein-digestibility-corrected amino acid score, PDCAAS) [169]. A factor responsible for the underutilization of pea protein is its low digestibility when compared to animal protein. A study compared the DIAAS of four dairy proteins and four plant proteins (pea protein concentrate, soybean protein isolate, soya flour, and whole grain wheat) in pigs [44]. The DIAAS values were calculated as recommended for PDCAAS, and the results showed greater PDCAAS-like values for the dairy proteins than for the plant proteins. Another study compared the nutritional quality of milk casein and pea protein isolate in 15 healthy humans, and the results showed significantly lower digestibility of pea leucine, valine, lysine, and phenylalanine [170]. However, the results showed that although pea protein had less DIAAS than milk casein (1.0 and 1.45, respectively), pea protein demonstrated the ability to meet all amino acid requirements. Also, the real ileal digestibility and net postprandial protein utilization (NPPU) of pea protein were not different from those of milk casein [170]. 

The presence of naturally occurring antinutritional materials such as antigenic proteins, protease inhibitors, α-amylase inhibitors, lectins, alkaloids, saponins, and tannins would reduce the digestibility of pea proteins and nutrient availability in the gut [171]. Other contributing factors could occur during heat or alkaline processing. Dehulling, soaking, germination, conventional/microwave cooking (e.g., boiling, roasting, or frying), and fermentation are methods commonly used to improve the palatability, digestibility, and bioavailability of pea protein by inactivation of the antinutritional materials. A comprehensive review of the effect of processing conditions and fractionation on pea protein and other pulses was conducted by Rivera del Rio et al. [172].

Another study evaluated the effect of different processing treatments on the antinutritional materials and digestibility of the same cultivar of yellow field peas grown around four different locations in Saskatchewan [173]. The result showed a significant reduction in the activity of trypsin inhibitors and tannin and increased digestibility in peas that were processed by conventional and microwave cooking and in germinated and roasted pea seeds. This is because tannins are highly labile and soluble and will leach out or degrade easily during processing. Also, completely dehulled seeds had lower tannin content (1.56 mg Ecat/g) than the native pea seeds (2.37 mg Ecat/g) because tannin content is predominant in the seed coat. However, dehulled seeds had a lower reduction in the activity of trypsin inhibitors compared with seeds processed by heat treatment and germination. Furthermore, fermentation has been used to reduce the presence of antinutritional materials in isolated pea protein. A study showed that 11 h fermentation of pea protein concentrate using *Lactobacillus plantarum* reduced the activity of protease inhibitors, but digestibility was reduced at the 11th hour from 67.0% to 54.6% due to a reduced score of sulfur amino acids [174]. On the contrary, Skalickova et al. [175] fermented pea flour for 72 h at 37 °C, and the result showed that digestibility was enhanced and there were improved levels of glutamine, cysteine, and methionine. Limiting the SCAA level in pea protein will influence digestibility, which is another reason for the lower digestibility in plant proteins than in animal proteins [10], and digestibility could further be reduced during fermentation [174]. The solution to maintaining the content of SCAAs in pea protein during fermentation will be the use of bacteria that have less impact on SCAAs. Improved digestibility was also shown to increase the bioaccessibility of manganese and iron [175].

### 4.7. Functional Gap between Laboratory-Prepared Pea Protein Isolates and Commercial Brands

The food industry is one of the highest consumers of water and energy, and for this sector to remain in business and maintain sustainable growth, the use of processing techniques that offer increased efficiency and reduced water and energy consumption must be employed [176]. However, most of the processing methods that fit this description are carried out at the bench scale, as discussed in the previous sections. For example, the most common protein extraction method and drying technique at the industrial scale are AE-IP and spray drying, respectively. However, studies have shown a functional divide between laboratory-prepared pea protein isolates and commercial brands. Burger et al. [121] compared the physicochemical properties of spray-dried pea protein isolates, and the result showed low solubility, high surface hydrophobicity, and high aggregate formation in four out of the five protein samples. AE-IP is a relatively cheap and easy method of extraction when compared with other methods because of the favorable outcome of high protein purity and yield. However, the limitations of this method include the loss of some functionalities due to denaturation during the solubilization step at pH 8–11 [177]. The authors employed high-pressure homogenization to break up the aggregates which reduced surface hydrophobicity and solubility. Other authors have reported the use of heat pretreatment coupled with membrane ultrafiltration to improve the physicochemical and functional properties of commercial pea protein isolates [96]. High temperatures used in spray drying have been shown to alter the spatial conformations of the protein and impair functionalities; these effects are not seen in proteins prepared by freeze drying [178]. Bridging the divide between commercial and laboratory-prepared pea protein isolates or concentrates would require scaling up the lab protocols/methods and/or carrying out pretreatment steps to improve the spatial conformations of the protein.

### 4.8. Potential Processing Technologies Not Yet Applied to Pea Protein

Our discourse so far has been based on processing technologies that have been applied in the extraction and structural modulation of pea protein. Under chemical treatments, techniques like glycation, acylation, phosphorylation, and pH shifting have been widely used in pea protein processing. Other common methods are the biological class (i.e., fermentation, enzymatic hydrolysis, and germination) and physical class (i.e., thermal, ultrasound, extrusion, ultrafiltration, cold plasma, high-pressure treatment, and irradiation). However, our search reveals some physical methods (i.e., hydrodynamic cavitation and cavitation jet technology) that have been used in the processing of soybean protein but have not been applied to pea protein processing. A study showed that the application of hydrodynamic cavitation (HC at 550 W for 0, 15, 30 min) to soybean glycinin aggregates dissociated the aggregates and enhanced the physicochemical and functional properties of the protein [179]. Another study showed that cavitation jet technology improved the solubility of protein in soymilk flour [180].

This literature review indicates a notable gap in research related to the utilization of pressure-assisted protein processing technologies to augment yield and functionality. A promising avenue for future investigation is the exploration of the impacts of high pressure (HP) and abrupt pressure changes in hydrodynamic cavitation (HC) on the yield and quality of pea protein, as both methodologies are harmonious with the wet fractionation technique. Additionally, HC technology can offer controlled heating, which is crucial for the inactivation of the lipoxygenase enzyme responsible for the oxidation of polyunsaturated fatty acids and resultant off-flavors in pea proteins. Initial studies have demonstrated the positive influence of HP treatment, within 50–125 MPa, on soybean protein extraction yield, with conditions for HP attainable through high-pressure homogenizers (HPH) at pressures exceeding 35 MPa [181]. However, the limited throughput capacity of HP techniques restricts their commercial viability. Conversely, HC does not require pervasive high pressure and possesses superior throughput capacity. The localized pressure drop in the cavitation zone generates intense turbulence, shear stress, shock waves, and cell disruption, facilitating an increased yield of bioactive compounds, including proteins, when small particle raw material is dispersed in the liquid flow. Martynenko et al. [182] provided an extensive description of the principles and apparatuses related to hydrodynamic cavitation. This environmentally green approach offers several benefits such as diminished energy, extraction time, and solvent usage and yields higher-quality protein ingredients [183]. Regrettably, the capabilities of HC in pea protein extraction remain largely untapped and warrant further exploration.

## 5. Food Applications 

Plant-based proteins like PPI are taking center stage in research and food formulations for several reasons (i.e., religious inclinations, cost, health, availability, nutritional, environmental sustainability, and functional properties). Using pea proteins as ingredients in food and beverage formulation was well captured in a review by Boukid et al. [184]. The functional properties of proteins such as solubility; water- and oil-holding capacity; and gelation, foaming, and emulsification properties are exploited for the formulation of various food products. Pea protein is reportedly the best-suited and most popular plant-based protein to replace animal proteins because of its cost efficiency and reduced adverse effects on health and the environment. One of the most utilized processing techniques in pea protein utilization is the extrusion cooking method using high moisture (HME; >40%) or low moisture (LME; <35%) to produce meat and seafood analogs or extruded puff snacks, respectively [185]. Pea protein flours, concentrates, and isolates have been widely used for the formulation of composite flours together with ingredients from cereals and other legumes or pulses to produce extruded food snacks [186,187,188,189]. These fortified extruded snacks exhibit superior nutritional quality (high protein and balanced amino acid content) compared to snacks extruded from cereals only [185]. Extruded snack balls made from pea flour (60–90%) and cereal flour (rice or wheat) were less appreciated for taste (based on the beany flavor imparted by pea) by consumers, especially at high pea concentration, but increased crispiness and puffiness were positive perceptions reported by consumers [190]. Crackers produced from dehulled oats and PPI (COP) showed superior textural and nutritional quality when compared with two commercial crackers [191]. For example, the protein contents of the COP and commercial crackers were 25 and 10%, respectively, and the COP crackers were chewier than the commercial brand. However, sensory analysis was not carried out to determine consumer acceptance.

Nowadays, there is a surge in the consumption of plant-protein-based meat or seafood analog products. Pea proteins can create HME fibrous-textured extrudates to mimic meat products [43,167,192]. Wheat protein has been the popular option for producing meat replacements due to the viscoelastic and rheological properties of gluten which acts as a binder and is used in composites with soybean protein isolates. Currently, gluten-free and hypoallergenic ingredients like peas, rice, oats, and other pulses are replacing the use of wheat and soybeans [192,193]. The incorporation of oat protein (30%) with pea protein improved the sensory characteristics of meat analogs [193], and trained panelists’ evaluation of vegetable patties showed that products from soybean protein had more favorable scores (taste and texture) than different pulse proteins, including pea protein [192]. However, no significant differences were observed in the taste and texture of veggie patties produced from pea, lentil, and fava bean proteins [192].

Research in the replacement of other protein sources with pea protein in the production of beverages is progressing and yielding positive outcomes. The application of different plant proteins (pea, soybean, rice, and almond) in fermented beverages showed that the product from pea protein exhibited the highest viscosity and coagulum strength with no syneresis [194]. Klost and Drusch [195] prepared a base formulation for plant-protein-based yogurt alternatives using 10% pea protein isolate with or without oil and fiber supplementation. However, sensory evaluation of the product was not reported. Partial replacement of milk protein by pea protein (0–40 g/100 g protein) and fermentation had no favorable outcome on sensory attributes of yogurt; an increase in the concentration of pea protein led to higher acidity, higher syneresis, and weak gels [196].

PPI has been widely used as a carrier in nanoencapsulation technology to preserve and transport labile nutrients and nutraceuticals. Akkam et al. [197] prepared pea protein nanoemulsions (10 mg/mL) with 1.0% (*w*/*w*) cholecalciferol/canola oil to improve the stability of vitamin D when fortified in food formulations such as milk and juices. Nanoemulsions that carried 20 μg vitamin D/mL exhibited superior water holding, foam, emulsification, and antioxidant properties when compared to the PPI prepared by ultrasound treatment [197]. The addition of the nanoemulsions and vitamin D did not alter the sensory properties of the juices and milk but enhanced the stability of vitamin D and nutritional value of the formulations, and consumer evaluation based on overall impression, aroma, consistency, and color was positive. Nanoparticles formed from a chitosan–PPI complex for the encapsulation of hyssop essential oil exhibited higher antioxidant activity than the free hyssop essential oil [198].

## 6. Concluding Remarks and Future Research Directions 

Yellow field pea is a high-protein crop with variations in chemical composition from different cultivars, and even when the same cultivar is grown in the same environment, the harvest and storage conditions are important factors. Extensive work has been carried out on optimizing the functional properties of pea proteins for applications in the food industry. The major challenges in using pea protein as an ingredient are the intrinsic and extrinsic factors (i.e., native form, processing, and storage). These lead to discrepancies and gaps in the functional properties and flavor output of the ingredient. Notably, laboratory-prepared field pea proteins maintain most of their native functionality, while commercial pea ingredients mainly have high degrees of denaturation because of conventional extraction methods like AE-IEP and spray drying conditions. Research has shown that the existing conventional extraction methods (dry and wet fractionation) do not provide optimal results (yield, purity, and functionality) alone and may be more effective when used as a hybrid model. Dry fractionation could produce protein concentrates with preserved native properties, but their purity is low (40–50% protein content), while protein isolates obtained from wet fractionation have high purity but are denatured with impaired functionalities. The purity of dry-fractionated proteins could be improved by ~15% using electrostatic methods in combination with or as an alternative to air classification. Milder wet or aqueous fractionation methods (e.g., enzyme-assisted, ultrafiltration) have been used either as a standalone technology or in combination with the AE-IEP method to produce proteins with superior yield, purity, and functionality when compared to conventional methods. A divide exists between the functional properties of laboratory-prepared and commercially prepared pea protein products because of the harsh extraction methods used for the latter. This gap can be effectively bridged using milder technologies (hybrid or standalone) to maximize yield, purity, and protein functionalities. In addition to the choice of the appropriate extraction method, research has shown that the use of chemical, physical, and enzymatic methods to modulate the complex conformation and improve the organoleptic and nutritional properties of pea protein have great potential to produce proteins with a high quality that is suitable for industrial use and consumer acceptability. This literature review indicates a notable gap in research related to the utilization of feedstock pretreatment technologies to enhance pea protein extraction in terms of yields and functionalities. For example, cavitation technology, based on ultrasonication or hydrodynamic pressure changes in the flow stream, is widely accepted for cell disruption. It has been applied in the extraction of soybean protein and should be explored for pea protein extraction. The authors are currently working on a project to utilize hydrodynamic cavitation for the pretreatment of yellow peas for protein extraction. The impact of abrupt pressure changes produced by hydrodynamic cavitation on the yield and quality of pea protein will be explored. The economic advantages of the pretreatment technologies and hybrid methods inspire industry interest in commercial implementation.

## Figures and Tables

**Table 1:** Proximate composition of isolated pea proteins from different cultivars.

Cultivar	Dry Matter (%)	Protein (%)	Ash (%)	Fat (%)	Protein Yield (g/kg)
Navarro	93.0 ± 0.0 ^ab^	83.5 ± 0.4 ^c^	5.3 ± 0.3 ^cd^	5.9 ± 0.0 ^c^	33.8
Dolores	93.5 ± 0.1 ^ab^	89.5 ± 0.2 ^a^	5.4 ± 0.1 ^cd^	4.7 ± 0.1 ^d^	54.4
Greenwich	93.8 ± 0.0 ^ab^	83.6 ± 0.4 ^c^	6.0 ± 0.6 ^c^	9.0 ± 0.2 ^a^	34.8
Bluetime	94.4 ± 0.0 ^a^	84.1 ± 0.0 ^bc^	6.4 ± 0.4 ^c^	8.4 ± 0.3 ^a^	42.2
Ostinato	94.1 ± 0.0 ^a^	86.0 ± 0.5 ^b^	7.6 ± 0.4 ^b^	7.1 ± 0.4 ^b^	38.6
Kalifa	93.0 ± 0.0 ^ab^	86.9 ± 0.9 ^b^	5.9 ± 0.1 ^c^	7.0 ± 0.5 ^b^	46.2
Salamanca	93.7 ± 0.6 ^ab^	85.0 ± 0.3 ^bc^	6.1 ± 1.0 ^c^	8.7 ± 0.6 ^a^	42.2
Florida	92.5 ± 0.0 ^b^	87.4 ± 1.1 ^b^	5.6 ± 0.1 ^cd^	7.4 ± 0.7 ^b^	59.2
RLPY 141091	93.4 ± 0.0 ^ab^	90.3 ± 0.0 ^a^	8.5 ± 0.7 ^a^	7.3 ± 0.8 ^b^	53.6
Orchestra	92.8 ± 0.3 ^b^	87.1 ± 0.1 ^b^	6.7 ± 1.1 ^c^	6.2 ± 0.9 ^c^	62.2
Astronaute	96.0 ± 0.2 ^a^	86.4 ± 0.1 ^b^	5.4 ± 0.1 ^cd^	7.8 ± 0.1 ^b^	42.1
Croft	92.5 ± 0.1 ^b^	86.7 ± 0.6 ^b^	6.2 ± 0.1 ^c^	7.8 ± 0.1 ^b^	47.3

^a–d^ Different letters indicate significant differences at the *p* ≤ 0.05 level for each column. Result expressed as mean ± standard deviation (*n* = 2) adapted from Garcia-Arteaga [17].

**Table 2:** Polypeptide composition of yellow field pea protein.

Classification	Content	Protein Fraction	Polypeptide	Svedberg Unit	Features	Author
Globulins	55–65%	Hexameric/quarternary legumin (300–600 kDa)	Six paired α and β (60–80 kDa)	11S	α and β subunits linked by disulfide linkage	Gueguen and Cerletti [26]; Lam et al. [18]; Tzitzikas et al. [27]
		Trimeric vicilin (175–180 kDa)	α, β, and γ (14–20 kDa)	7S	Non-covalent bonds between subunits and glycosylation	Chang et al. [28]; Kaur Dhaliwal et al. [29]
		Trimeric convicilin (210 kDa)	~70 kDa	8S	80% amino acid homology with 7S	Kaur Dhaliwal et al. [29]; Mertens et al. [30]
Albumins	18–25%	Pea albumins	PA1a (5.8 kDa)	2S	53 amino acids and high Cys	Barbana and Boye [31]; De Santis et al. [32]; Kornet et al. [33]; Park et al. [25]
			PA1b (4.0 kDa)	2S	37 amino acids and high Cys	
		Lectins	n/a	n/a	n/a	
		Lipoxygenase	90–100 kDa	n/a	n/a	
		Protease inhibitors		n/a	n/a	
		Natural pigments (anthocyanins and tannins)		n/a	n/a	
Prolamin	4–5%	n/a	n/a	n/a	High Glu and Pro	Adebiyi and Aluko [34]
Glutelin	3–4%	n/a	n/a	n/a	n/a	

## Data Availability

No new data was created for this work. Review was majorly based on peer reviewed articles from scientists.

## References

[1] Rasskazova I., Kirse-Ozolina A. (2020). Field Pea *Pisum sativum* L. as a Perspective Ingredient for Vegan Foods: A Review. Res. Rural. Dev..

[2] Mudryj A.N., Yu N., Aukema H.M. (2014). Nutritional and Health Benefits of Pulses. Appl. Physiol. Nutr. Metab..

[3] Pasiakos S., Agarwal S., Lieberman H., Fulgoni V. (2015). Sources and Amounts of Animal, Dairy, and Plant Protein Intake of US Adults in 2007–2010. Nutrients.

[4] Barac M., Cabrilo S., Pesic M., Stanojevic S., Zilic S., Macej O., Ristic N. (2010). Profile and Functional Properties of Seed Proteins from Six Pea (*Pisum sativum*) Genotypes. Int. J. Mol. Sci..

[5] Choudhury D., Singh S., Seah J.S.H., Yeo D.C.L., Tan L.P. (2020). Commercialization of Plant-Based Meat Alternatives. Trends Plant Sci..

[6] Pelgrom P.J.M., Boom R.M., Schutyser M.A.I. (2015). Food Hydrocolloids Functional Analysis of Mildly Refined Fractions from Yellow Pea. Food Hydrocoll..

[7] Pelgrom P.J.M., Vissers A.M., Boom R.M., Schutyser M.A.I. (2013). Dry Fractionation for Production of Functional Pea Protein Concentrates. Food Res. Int..

[8] Pelgrom P.J.M., Wang J., Boom R.M., Schutyser M.A.I. (2015). Pre- and Post-Treatment Enhance the Protein Enrichment from Milling and Air Classification of Legumes. J. Food Eng..

[9] Xing Q., Utami D.P., Demattey M.B., Kyriakopoulou K., de Wit M., Boom R.M., Schutyser M.A.I. (2020). A Two-Step Air Classification and Electrostatic Separation Process for Protein Enrichment of Starch-Containing Legumes. Innov. Food Sci. Emerg. Technol..

[10] Gorissen S.H.M., Crombag J.J.R., Senden J.M.G., Waterval W.A.H., Bierau J., Verdijk L.B., van Loon L.J.C. (2018). Protein Content and Amino Acid Composition of Commercially Available Plant-Based Protein Isolates. Amino Acids.

[11] Malafronte L., Ruoff D., Gunes D.Z., Lequeux F., Schmitt C., Windhab E.J. (2019). Morphology Development in Single Drop Drying for Native and Aggregated Whey Protein Dispersions. Colloids Surf. A Physicochem. Eng. Asp..

[12] Field Pea Breeding at Agriculture and Agri-Food Canada. https://www.manitobapulse.ca/2020/12.

[13] Pulse and Soybean Variety Guide. https://www.manitobapulse.ca/production/variety-evaluation-guide/.

[14] Maharjan P., Penny J., Partington D.L., Panozzo J.F. (2019). Genotype and Environment Effects on the Chemical Composition and Rheological Properties of Field Peas. J. Sci. Food Agric..

[15] Millar K.A., Gallagher E., Burke R., McCarthy S., Barry-Ryan C. (2019). Proximate Composition and Anti-Nutritional Factors of Fava-Bean (*Vicia faba*), Green-Pea and Yellow-Pea (*Pisum sativum*) Flour. J. Food Compos. Anal..

[16] Kornet R., Veenemans J., Venema P., van der Goot A.J., Meinders M., Sagis L., van der Linden E. (2021). Less Is More: Limited Fractionation Yields Stronger Gels for Pea Proteins. Food Hydrocoll..

[17] García Arteaga V., Kraus S., Schott M., Muranyi I., Schweiggert-Weisz U., Eisner P. (2021). Screening of Twelve Pea (*Pisum sativum* L.) Cultivars and Their Isolates Focusing on the Protein Characterization, Functionality, and Sensory Profiles. Foods.

[18] Lam A.C.Y., Warkentin T.D., Tyler R.T., Nickerson M.T. (2017). Physicochemical and Functional Properties of Protein Isolates Obtained from Several Pea Cultivars. Cereal Chem. J..

[19] Food Safety Authority of Ireland (2021). Information on Nutrition and Health Claims.

[20] Nikolopoulou D., Grigorakis K., Stasini M., Alexis M.N., Iliadis K. (2007). Differences in Chemical Composition of Field Pea (*Pisum sativum*) Cultivars: Effects of Cultivation Area and Year. Food Chem..

[21] Gao Z., Shen P., Lan Y., Cui L., Ohm J.-B., Chen B., Rao J. (2020). Effect of Alkaline Extraction pH on Structure Properties, Solubility, and Beany Flavor of Yellow Pea Protein Isolate. Food Res. Int..

[22] Pedrosa M.M., Varela A., Domínguez-Timón F., Tovar C.A., Moreno H.M., Borderías A.J., Díaz M.T. (2020). Comparison of Bioactive Compounds Content and Techno-Functional Properties of Pea and Bean Flours and Their Protein Isolates. Plant Foods Hum. Nutr. (Dordr.).

[23] Stone A.K., Karalash A., Tyler R.T., Warkentin T.D., Nickerson M.T. (2015). Functional Attributes of Pea Protein Isolates Prepared Using Different Extraction Methods and Cultivars. Food Res. Int..

[24] Dziuba J., Szerszunowicz I., Nałecz D., Dziuba M. (2014). Proteomic Analysis of Albumin and Globulin Fractions of Pea (*Pisum sativum* L.) Seeds. Acta Sci. Polonorum. Technol. Aliment..

[25] Park S.J., Kim T.W., Baik B.-K. (2010). Relationship between Proportion and Composition of Albumins, and In Vitro Protein Digestibility of Raw and Cooked Pea Seeds (*Pisum sativum* L.). J. Sci. Food Agric..

[26] Gueguen J., Cerletti P., Hudson B.J.F. (1994). Proteins of Some Legume Seeds: Soybean, Pea, Fababean and Lupin BT—New and Developing Sources of Food Proteins.

[27] Tzitzikas E.N., Vincken J.-P., de Groot J., Gruppen H., Visser R.G.F. (2006). Genetic Variation in Pea Seed Globulin Composition. J. Agric. Food Chem..

[28] Chang L., Lan Y., Bandillo N., Ohm J.-B., Chen B., Rao J. (2022). Plant Proteins from Green Pea and Chickpea: Extraction, Fractionation, Structural Characterization and Functional Properties. Food Hydrocoll..

[29] Kaur Dhaliwal S., Salaria P., Kaushik P. (2021). Pea Seed Proteins: A Nutritional and Nutraceutical Update. Grain and Seed Proteins Functionality.

[30] Mertens C., Dehon L., Bourgeois A., Verhaeghe-Cartrysse C., Blecker C. (2012). Agronomical Factors Influencing the Legumin/Vicilin Ratio in Pea (*Pisum sativum* L.) Seeds. J. Sci. Food Agric..

[31] Barbana C., Boye J.I. (2013). In Vitro Protein Digestibility and Physico-Chemical Properties of Flours and Protein Concentrates from Two Varieties of Lentil (*Lens culinaris*). Food Funct..

[32] De Santis M.A., Rinaldi M., Menga V., Codianni P., Giuzio L., Fares C., Flagella Z. (2021). Influence of Organic and Conventional Farming on Grain Yield and Protein Composition of Chickpea Genotypes. Agronomy.

[33] Kornet R., Yang J., Venema P., van der Linden E., Sagis L.M.C. (2022). Optimizing Pea Protein Fractionation to Yield Protein Fractions with a High Foaming and Emulsifying Capacity. Food Hydrocoll..

[34] Adebiyi A.P., Aluko R.E. (2011). Functional Properties of Protein Fractions Obtained from Commercial Yellow Field Pea (*Pisum sativum* L.) Seed Protein Isolate. Food Chem..

[35] Tang C., Sun X., Yin S. (2009). Food Hydrocolloids Physicochemical, Functional and Structural Properties of Vicilin-Rich Protein Isolates from Three Phaseolus Legumes: Effect of Heat Treatment. Food Hydrocoll..

[36] Zhao H., Shen C., Wu Z., Zhang Z., Xu C. (2020). Comparison of Wheat, Soybean, Rice, and Pea Protein Properties for Effective Applications in Food Products. J. Food Biochem..

[37] Sui X., Zhang T., Jiang L. (2021). Soy Protein: Molecular Structure Revisited and Recent Advances in Processing Technologies. Annu. Rev. Food Sci. Technol..

[38] Ibáñez M.A., de Blas C., Cámara L., Mateos G.G. (2020). Chemical Composition, Protein Quality and Nutritive Value of Commercial Soybean Meals Produced from Beans from Different Countries: A Meta-Analytical Study. Anim. Feed Sci. Technol..

[39] Damodaran S., Paraf A. (2017). Food Proteins and Their Applications.

[40] Zhu X., Ning C., Li S., Xu P., Zheng Y., Zhou C. (2018). Effects of L-lysine/L-arginine on the Emulsion Stability, Textural, Rheological and Microstructural Characteristics of Chicken Sausages. Int. J. Food Sci. Technol..

[41] Martínez-Villaluenga C., Gulewicz P., Frias J., Gulewicz K., Vidal-Valverde C. (2008). Assessment of Protein Fractions of Three Cultivars of *Pisum sativum* L.: Effect of Germination. Eur. Food Res. Technol..

[42] Emkani M., Oliete B., Saurel R. (2021). Pea Protein Extraction Assisted by Lactic Fermentation: Impact on Protein Profile and Thermal Properties. Foods.

[43] Osen R., Toelstede S., Eisner P., Schweiggert-Weisz U. (2015). Effect of High Moisture Extrusion Cooking on Protein-Protein Interactions of Pea (*Pisum sativum* L.) Protein Isolates. Int. J. Food Sci. Technol..

[44] Mathai J.K., Liu Y., Stein H.H. (2017). Values for Digestible Indispensable Amino Acid Scores (DIAAS) for Some Dairy and Plant Proteins May Better Describe Protein Quality than Values Calculated Using the Concept for Protein Digestibility-Corrected Amino Acid Scores (PDCAAS). Br. J. Nutr..

[45] Rutherfurd S.M., Fanning A.C., Miller B.J., Moughan P.J. (2015). Protein Digestibility-Corrected Amino Acid Scores and Digestible Indispensable Amino Acid Scores Differentially Describe Protein Quality in Growing Male Rats. J. Nutr..

[46] van den Berg L.A., Mes J.J., Mensink M., Wanders A.J. (2022). Protein Quality of Soy and the Effect of Processing: A Quantitative Review. Front. Nutr..

[47] Nosworthy M.G., Franczyk A.J., Medina G., Neufeld J., Appah P., Utioh A., Frohlich P., House J.D. (2017). Effect of Processing on the In Vitro and In Vivo Protein Quality of Yellow and Green Split Peas (*Pisum sativum*). J. Agric. Food Chem..

[48] Trindler C., Annika Kopf-Bolanz K., Denkel C. (2022). Aroma of Peas, Its Constituents and Reduction Strategies—Effects from Breeding to Processing. Food Chem..

[49] Wang Y., Guldiken B., Tulbek M., House J.D., Nickerson M. (2020). Impact of Alcohol Washing on the Flavour Profiles, Functionality and Protein Quality of Air Classified Pea Protein Enriched Flour. Food Res. Int..

[50] Hanan E., Rudra S.G., Sharma V., Sagar V.R., Sehgal S. (2021). Pea Pod Powder to Enhance the Storage Quality of Buckwheat Bread. Vegetos.

[51] Cui L., Kimmel J., Zhou L., Rao J., Chen B. (2020). Identification of Extraction pH and Cultivar Associated Aromatic Compound Changes in Spray Dried Pea Protein Isolate Using Untargeted and Targeted Metabolomic Approaches. J. Agric. Food Res..

[52] García Arteaga V., Leffler S., Muranyi I., Eisner P., Schweiggert-Weisz U. (2021). Sensory Profile, Functional Properties and Molecular Weight Distribution of Fermented Pea Protein Isolate. Curr. Res. Food Sci..

[53] Vara-Ubol S., Chambers E., Chambers D.H. (2004). Sensory Characterisitcs of Chemical Componuds Potentially Associated with Beany Aroma in Foods. J. Sens. Stud..

[54] Yu H., Liu R., Hu Y., Xu B. (2017). Flavor Profiles of Soymilk Processed with Four Different Processing Technologies and 26 Soybean Cultivars Grown in China. Int. J. Food Prop..

[55] Lv Y.-C., Song H.-L., Li X., Wu L., Guo S.-T. (2011). Influence of Blanching and Grinding Process with Hot Water on Beany and Non-Beany Flavor in Soymilk. J. Food Sci..

[56] Liu J., Klebach M., Visser M., Hofman Z. (2019). Amino Acid Availability of a Dairy and Vegetable Protein Blend Compared to Single Casein, Whey, Soy, and Pea Proteins: A Double-Blind, Cross-Over Trial. Nutrients.

[57] Joint FAO/WHO/UNU (2007). Expert Consultation on Protein and Amino Acid Requirements in Human. Protein and Amino Acid Requirements in Human Nutrition: Report of a Joint FAO/WHO/UNU Expert Consultation.

[58] Reynaud Y., Buffière C., Cohade B., Vauris M., Liebermann K., Hafnaoui N., Lopez M., Souchon I., Dupont D., Rémond D. (2021). True Ileal Amino Acid Digestibility and Digestible Indispensable Amino Acid Scores (DIAASs) of Plant-Based Protein Foods. Food Chem..

[59] Shi Y. (2020). Reducing Off-Flavour in Plant Protein Isolates by Lactic Acid Fermentation. Ph.D. Thesis.

[60] Ma Z., Boye J.I., Azarnia S., Simpson B.K. (2016). Volatile Flavor Profile of Saskatchewan Grown Pulses as Affected by Different Thermal Processing Treatments. Int. J. Food Prop..

[61] Schindler S., Zelena K., Krings U., Bez J., Eisner P., Berger R.G. (2012). Improvement of the Aroma of Pea (*Pisum sativum*) Protein Extracts by Lactic Acid Fermentation. Food Biotechnol..

[62] El Youssef C., Bonnarme P., Fraud S., Péron A.-C., Helinck S., Landaud S. (2020). Sensory Improvement of a Pea Protein-Based Product Using Microbial Co-Cultures of Lactic Acid Bacteria and Yeasts. Foods.

[63] Yang M., Li N., Tong L., Fan B., Wang L., Wang F., Liu L. (2021). Comparison of Physicochemical Properties and Volatile Flavor Compounds of Pea Protein and Mung Bean Protein-Based Yogurt. LWT.

[64] Guldiken B., Green R., Nickerson M.T. (2021). The Impact of Different Adsorbents on Flavour Characteristics of a Lentil Protein Isolate. Eur. Food Res. Technol..

[65] Li C., Chen X., Jin Z., Gu Z., Rao J., Chen B. (2021). Physicochemical Property Changes and Aroma Differences of Fermented Yellow Pea Flours: Role of *Lactobacilli* and Fermentation Time. Food Funct..

[66] Frohlich P., Young G., Borsuk Y., Sigvaldson M., Bourré L., Sopiwnyk E. (2021). Influence of Premilling Thermal Treatments of Yellow Peas, Navy Beans, and Fava Beans on the Flavor and End-product Quality of Tortillas and Pitas. Cereal Chem..

[67] Zha F., Yang Z., Rao J., Chen B. (2019). Gum Arabic-Mediated Synthesis of Glyco-Pea Protein Hydrolysate via Maillard Reaction Improves Solubility, Flavor Profile, and Functionality of Plant Protein. J. Agric. Food Chem..

[68] Zhou X., Cui H., Zhang Q., Hayat K., Yu J., Hussain S., Tahir M.U., Zhang X., Ho C.-T. (2021). Taste Improvement of Maillard Reaction Intermediates Derived from Enzymatic Hydrolysates of Pea Protein. Food Res. Int..

[69] Benavides-Paz Y.L., Ismail B.P., Reineccius G.A. (2022). Monitoring the Aroma Profile During the Production of a Pea Protein Isolate by Alkaline Solubilization Coupled with Isoelectric Precipitation. ACS Food Sci. Technol..

[70] Yang J., Zamani S., Liang L., Chen L. (2021). Extraction Methods Significantly Impact Pea Protein Composition, Structure and Gelling Properties. Food Hydrocoll..

[71] Zhu H.-G., Tang H.-Q., Cheng Y.-Q., Li Z.-G., Tong L.-T. (2021). Novel Electromagnetic Separation Technology for the Production of Pea Protein Concentrate. Innov. Food Sci. Emerg. Technol..

[72] Saldanha do Carmo C., Silventoinen P., Nordgård C.T., Poudroux C., Dessev T., Zobel H., Holtekjølen A.K., Draget K.I., Holopainen-Mantila U., Knutsen S.H. (2020). Is Dehulling of Peas and Faba Beans Necessary Prior to Dry Fractionation for the Production of Protein- and Starch-Rich Fractions? Impact on Physical Properties, Chemical Composition and Techno-Functional Properties. J. Food Eng..

[73] Fenn D., Wang N., Maximiuk L. (2021). Physicochemical, Anti-nutritional, and Functional Properties of Air-classified Protein Concentrates from Commercially Grown Canadian Yellow Pea (*Pisum sativum*) Varieties with Variable Protein Levels. Cereal Chem..

[74] Politiek R.G.A., He S., Wilms P.F.C., Keppler J.K., Bruins M.E., Schutyser M.A.I. (2023). Effect of Relative Humidity on Milling and Air Classification Explained by Particle Dispersion and Flowability. J. Food Eng..

[75] Schlangen M., Taghian Dinani S., Schutyser M.A.I., van der Goot A.J. (2022). Dry Fractionation to Produce Functional Fractions from Mung Bean, Yellow Pea and Cowpea Flour. Innov. Food Sci. Emerg. Technol..

[76] Möller A.C., Li J., van der Goot A.J., van der Padt A. (2022). A Water-Only Process to Fractionate Yellow Peas into Its Constituents. Innov. Food Sci. Emerg. Technol..

[77] Möller A.C., van der Padt A., van der Goot A.J. (2022). Influence of the Fractionation Method on the Protein Composition and Functional Properties. Innov. Food Sci. Emerg. Technol..

[78] Tirgarian B., Farmani J., Milani J.M. (2019). Enzyme-Assisted Aqueous Extraction of Oil and Protein Hydrolysate from Sesame Seed. J. Food Meas. Charact..

[79] García Arteaga V., Apéstegui Guardia M., Muranyi I., Eisner P., Schweiggert-Weisz U. (2020). Effect of Enzymatic Hydrolysis on Molecular Weight Distribution, Techno-Functional Properties and Sensory Perception of Pea Protein Isolates. Innov. Food Sci. Emerg. Technol..

[80] Pulse Canada. https://pulsecanada.com/processing/processing-technology.

[81] Fernando S. (2021). Production of Protein-Rich Pulse Ingredients through Dry Fractionation: A Review. Food Sci. Technol..

[82] Joyner J.J., Yadav B.K. (2013). Microwave Assisted Dehulling of Black Gram (*Vigna Mungo* L). J. Food Sci. Technol..

[83] Sunil C.K., Chidanand D.V., Manoj D., Choudhary P., Rawson A. (2018). Effect of Ultrasound Treatment on Dehulling Efficiency of Blackgram. J. Food Sci. Technol..

[84] Wang J., Zhao J., de Wit M., Boom R.M., Schutyser M.A.I. (2016). Lupine Protein Enrichment by Milling and Electrostatic Separation. Innov. Food Sci. Emerg. Technol..

[85] Tabtabaei S., Vitelli M., Rajabzadeh A.R., Legge R.L. (2017). Analysis of Protein Enrichment during Single- and Multi-Stage Tribo-Electrostatic Bioseparation Processes for Dry Fractionation of Legume Flour. Sep. Purif. Technol..

[86] Zhang J., Liu L., Liu H., Yoon A., Rizvi S.S.H., Wang Q. (2019). Changes in Conformation and Quality of Vegetable Protein during Texturization Process by Extrusion. Crit. Rev. Food Sci. Nutr..

[87] Schutyser M.A.I., Pelgrom P.J.M., van der Goot A.J., Boom R.M. (2015). Dry Fractionation for Sustainable Production of Functional Legume Protein Concentrates. Trends Food Sci. Technol..

[88] Jafari M., Rajabzadeh A.R., Tabtabaei S., Marsolais F., Legge R.L. (2016). Physicochemical Characterization of a Navy Bean (*Phaseolus vulgaris*) Protein Fraction Produced Using a Solvent-Free Method. Food Chem..

[89] Möller A.C., van der Padt A., van der Goot A.J. (2021). From Raw Material to Mildly Refined Ingredient—Linking Structure to Composition to Understand Fractionation Processes. J. Food Eng..

[90] Ramirez J. (2017). Ultrafiltration: Methods, Applications and Insights.

[91] Charcosset C. (2012). 2-Ultrafiltration. Membrane Processes in Biotechnology and Pharmaceutics.

[92] Etzel M.R., Arunkumar A. (2015). Dairy Protein Fractionation and Concentration Using Charged Ultrafiltration Membranes.

[93] Metsämuuronen S., Mänttäri M., Nyström M. (2011). Comparison of Analysis Methods for Protein Concentration and Its Use in UF Fractionation of Whey. Desalination.

[94] Ratnaningsih E., Reynard R., Khoiruddin K., Wenten I.G., Boopathy R. (2021). Recent Advancements of UF-Based Separation for Selective Enrichment of Proteins and Bioactive Peptides—A Review. Appl. Sci..

[95] Boye J.I., Aksay S., Roufik S., Ribéreau S., Mondor M., Farnworth E., Rajamohamed S.H. (2010). Comparison of the Functional Properties of Pea, Chickpea and Lentil Protein Concentrates Processed Using Ultrafiltration and Isoelectric Precipitation Techniques. Food Res. Int..

[96] Asen N.D., Aluko R.E. (2022). Physicochemical and Functional Properties of Membrane-Fractionated Heat-Induced Pea Protein Aggregates. Front. Nutr..

[97] Berghout J.A.M., Marmolejo-Garcia C., Berton-Carabin C.C., Nikiforidis C.V., Boom R.M., van der Goot A.J. (2015). Aqueous Fractionation Yields Chemically Stable Lupin Protein Isolates. Food Res. Int..

[98] Zhu H.-G., Tang H.-Q., Cheng Y.-Q., Li Z.-G., Tong L.-T. (2021). Potential of Preparing Meat Analogue by Functional Dry and Wet Pea (*Pisum sativum*) Protein Isolate. LWT.

[99] Görgüç A., Özer P., Yılmaz F.M. (2020). Simultaneous Effect of Vacuum and Ultrasound Assisted Enzymatic Extraction on the Recovery of Plant Protein and Bioactive Compounds from Sesame Bran. J. Food Compos. Anal..

[100] Yang J., Liu G., Zeng H., Chen L. (2018). Effects of High Pressure Homogenization on Faba Bean Protein Aggregation in Relation to Solubility and Interfacial Properties. Food Hydrocoll..

[101] Amat T., Assifaoui A., Buczkowski J., Silva J.V.C., Schmitt C., Saurel R. (2024). Interplay between Soluble and Insoluble Protein/Calcium/Phytic Acid Complexes in Dispersions of Faba Bean and Pea Protein Concentrates around Neutral pH. Food Hydrocoll..

[102] Awosika T., Aluko R.E. (2019). Enzymatic Pea Protein Hydrolysates Are Active Trypsin and Chymotrypsin Inhibitors. Foods.

[103] Awosika T.O., Aluko R.E. (2019). Inhibition of the in Vitro Activities of A-amylase, A-glucosidase and Pancreatic Lipase by Yellow Field Pea (*Pisum sativum* L.) Protein Hydrolysates. Int. J. Food Sci. Tech..

[104] Asen N.D., Aluko R.E. (2023). Effect of Heat Treatment on Yellow Field Pea (*Pisum sativum*) Protein Concentrate Coupled with Membrane Ultrafiltration on Emulsification Properties of the Isolated >50 kDa Proteins. Membranes.

[105] Hansen L., Bu F., Ismail B.P. (2022). Structure-Function Guided Extraction and Scale-Up of Pea Protein Isolate Production. Foods.

[106] Aluko R.E. (2015). Antihypertensive Peptides from Food Proteins. Annu. Rev. Food Sci. Technol..

[107] Sánchez-Velázquez O.A., Manzanilla-Valdez M.L., Wang Y., Mondor M., Hernández-Álvarez A.J., Hernández-Álvarez A.J., Mondor M., Nosworthy M.G. (2023). Micellar Precipitation and Reverse Micelle Extraction of Plant Proteins. Green Protein Processing Technologies from Plants: Novel Extraction and Purification Methods for Product Development.

[108] Mondor M., Hernandez-alvarez A.J. (2022). Processing Technologies to Produce Plant Protein Concentrates and Isolates. Plant Protein Foods.

[109] Tanger C., Engel J., Kulozik U. (2020). Influence of Extraction Conditions on the Conformational Alteration of Pea Protein Extracted from Pea Flour. Food Hydrocoll..

[110] Boye J., Zare F., Pletch A. (2010). Pulse Proteins: Processing, Characterization, Functional Properties and Applications in Food and Feed. Food Res. Int..

[111] Sun X.D., Arntfield S.D. (2011). Gelation Properties of Salt-Extracted Pea Protein Isolate Induced by Heat Treatment: Effect of Heating and Cooling Rate. Food Chem..

[112] Geerts M.E.J., Mienis E., Nikiforidis C.V., van der Padt A., van der Goot A.J. (2017). Mildly Refined Fractions of Yellow Peas Show Rich Behaviour in Thickened Oil-in-Water Emulsions. Innov. Food Sci. Emerg. Technol..

[113] Görgüç A., Özer P., Yılmaz F.M. (2020). Microwave-assisted Enzymatic Extraction of Plant Protein with Antioxidant Compounds from the Food Waste Sesame Bran: Comparative Optimization Study and Identification of Metabolomics Using LC/Q-TOF/MS. J. Food Process. Preserv..

[114] Pojić M., Mišan A., Tiwari B. (2018). Eco-Innovative Technologies for Extraction of Proteins for Human Consumption from Renewable Protein Sources of Plant Origin. Trends Food Sci. Technol..

[115] Kumar M., Tomar M., Potkule J., Verma R., Punia S., Mahapatra A., Belwal T., Dahuja A., Joshi S., Berwal M.K. (2021). Advances in the Plant Protein Extraction: Mechanism and Recommendations. Food Hydrocoll..

[116] Sari Y.W., Bruins M.E., Sanders J.P.M. (2013). Enzyme Assisted Protein Extraction from Rapeseed, Soybean, and Microalgae Meals. Ind. Crops Prod..

[117] Liu J., Gasmalla M.A.A., Li P., Yang R. (2016). Enzyme-Assisted Extraction Processing from Oilseeds: Principle, Processing and Application. Innov. Food Sci. Emerg. Technol..

[118] Das R. (2015). Multienzyme Modification of Hemp Protein for Functional Peptides Synthesis. J. Food Process..

[119] Schmidt F., Blankart M., Wanger J., Scharfe M., Scheuerer T., Hinrichs J. (2022). Upscaling of Alkaline Pea Protein Extraction from Dry Milled and Pre-Treated Peas from Laboratory to Pilot Scale: Optimization of Process Parameters for Higher Protein Yields. J. Food Meas. Charact..

[120] Karaca A.C., Low N., Nickerson M. (2011). Emulsifying Properties of Canola and Flaxseed Protein Isolates Produced by Isoelectric Precipitation and Salt Extraction. Food Res. Int..

[121] Burger T.G., Singh I., Mayfield C., Baumert J.L., Zhang Y. (2021). Comparison of Physicochemical and Emulsifying Properties of Commercial Pea Protein Powders. J. Sci. Food Agric..

[122] Aryee A.N.A., Agyei D., Udenigwe C.C., Yada R. (2018). Impact of Selected Process Parameters on Solubility and Heat Stability of Pea Protein Isolate. Proteins in Food Processing.

[123] Tang X., Shen Y., Zhang Y., Schilling M.W., Li Y. (2021). Parallel Comparison of Functional and Physicochemical Properties of Common Pulse Proteins. LWT.

[124] Shevkani K., Singh N. (2015). Relationship between Protein Characteristics and Film-Forming Properties of Kidney Bean, Field Pea and Amaranth Protein Isolates. Int. J. Food Sci. Technol..

[125] Pelegrine D.H.G., Gasparetto C.A. (2005). Whey Proteins Solubility as Function of Temperature and pH. LWT Food Sci. Technol..

[126] Kimura A., Fukuda T., Zhang M., Motoyama S., Maruyama N., Utsumi S. (2008). Comparison of Physicochemical Properties of 7S and 11S Globulins from Pea, Fava Bean, Cowpea, and French Bean with Those of Soybean—French Bean 7S Globulin Exhibits Excellent Properties. J. Agric. Food Chem..

[127] Liang H.-N., Tang C.-H. (2013). pH-Dependent Emulsifying Properties of Pea (*Pisum sativum* L.) Proteins. Food Hydrocoll..

[128] Hall A.E., Moraru C.I. (2021). Structure and Function of Pea, Lentil and Faba Bean Proteins Treated by High Pressure Processing and Heat Treatment. LWT.

[129] Zhao S., Huang Y., McClements D.J., Liu X., Wang P., Liu F. (2021). Improving Pea Protein Functionality by Combining High-Pressure Homogenization with an Ultrasound-Assisted Maillard Reaction. Food Hydrocoll..

[130] Zhi Z., Yan L., Li H., Dewettinck K., Van der Meeren P., Liu R., Van Bockstaele F. (2021). A Combined Approach for Modifying Pea Protein Isolate to Greatly Improve Its Solubility and Emulsifying Stability. Food Chem..

[131] Shevkani K., Singh N., Kaur A., Chand J. (2015). Food Hydrocolloids Structural and Functional Characterization of Kidney Bean and Field Pea Protein Isolates: A Comparative Study. Food Hydrocoll..

[132] Aryee A.N.A., Boye J.I. (2017). Comparative Study of the Effects of Processing on the Nutritional, Physicochemical and Functional Properties of Lentil. J. Food Process. Preserv..

[133] Nieto Nieto T.V., Wang Y., Ozimek L., Chen L. (2016). Improved Thermal Gelation of Oat Protein with the Formation of Controlled Phase-Separated Networks Using Dextrin and Carrageenan Polysaccharides. Food Res. Int..

[134] Zhan F., Tang X., Sobhy R., Li B., Chen Y. (2021). Structural and Rheology Properties of Pea Protein Isolate-stabilised Emulsion Gel: Effect of Crosslinking with Transglutaminase. Int. J. Food Sci. Technol..

[135] Li Y., Tian Y., Deng L., Dai T., Liu C., Chen J. (2024). High Energy Media Mill Modified Pea Dietary Fiber: Physicochemical Property and Its Mechanism in Stabilizing Pea Protein Beverage. Food Hydrocoll..

[136] Erdoğdu Ö., Görgüç A., Yılmaz F.M. (2023). Functionality Enhancement of Pea Protein Powder via High-Intensity Ultrasound: Screening in-Vitro Digestion, o/w Emulsion Properties and Testing in Gluten-Free Bread. Plant Foods Hum. Nutr..

[137] Tan J.X., Tan C.-C., Dharmawan J., Leong S.S. (2023). Effects of Ethanol Washing on Off-Flavours Removal and Protein Functionalities of Pea Protein Concentrate. Food Bioprod. Process..

[138] Batbayar B., Kryachko Y., Nickerson M.T., Korber D.R., Tanaka T. (2023). Solid-State and Submerged Fermentation Effects on Functional Properties of Pea Protein-Enriched Flour. Cereal Chem..

[139] Zhao L., Chen M.-H., Bi X., Du J. (2023). Physicochemical Properties, Structural Characteristics and In Vitro Digestion of Brown Rice–Pea Protein Isolate Blend Treated by Microbial Transglutaminase. Food Hydrocoll..

[140] Tang Y.R., Stone A.K., Wang Y., Jafarian Z., Zhou L., Kimmel J., House J.D., Tanaka T., Nickerson M.T. (2023). Effects of Enzyme Treatments on the Functionality of Commercial Pea and Pea Blended Protein Ingredients. Food Biosci..

[141] Zhao P., Li N., Chen L., Guo Y., Huang Y., Tong L., Wang L., Fan B., Wang F., Liu L. (2023). Effects of Oat β-Glucan on the Textural and Sensory Properties of Low-Fat Set Type Pea Protein Yogurt. Molecules.

[142] Zayas J.F. (1997). Foaming Properties of Proteins. Functionality of Proteins in Food.

[143] Kristensen H.T., Denon Q., Tavernier I., Gregersen S.B., Hammershøj M., Van der Meeren P., Dewettinck K., Dalsgaard T.K. (2021). Improved Food Functional Properties of Pea Protein Isolate in Blends and Co-Precipitates with Whey Protein Isolate. Food Hydrocoll..

[144] Rullier B., Novales B., Axelos M.A.V. (2008). Effect of Protein Aggregates on Foaming Properties of β-Lactoglobulin. Colloids Surf. A Physicochem. Eng. Asp..

[145] Du M., Xie J., Gong B., Xu X., Tang W., Li X., Li C., Xie M. (2018). Extraction, Physicochemical Characteristics and Functional Properties of Mung Bean Protein. Food Hydrocoll..

[146] Malomo S.A., He R., Aluko R.E. (2014). Structural and Functional Properties of Hemp Seed Protein Products. J. Food Sci..

[147] Taherian A.R., Mondor M., Labranche J., Drolet H., Ippersiel D., Lamarche F. (2011). Comparative Study of Functional Properties of Commercial and Membrane Processed Yellow Pea Protein Isolates. Food Res. Int..

[148] Chao D., Aluko R.E. (2018). Modification of the Structural, Emulsifying, and Foaming Properties of an Isolated Pea Protein by Thermal Pretreatment. CyTA J. Food.

[149] Saldanha do Carmo C., Nunes A.N., Silva I., Maia C., Poejo J., Ferreira-Dias S., Nogueira I., Bronze R., Duarte C.M.M. (2016). Formulation of Pea Protein for Increased Satiety and Improved Foaming Properties. RSC Adv..

[150] Chang L., Chen B., Rao J. (2023). Synergistic Effect of pH-Shift and Controlled Heating on Improving Foaming Properties of Pea Vicilin and Its Adsorption Behavior at the Air-Water Interface. Food Hydrocoll..

[151] Xie J., Huang W., Wu X. (2023). Effects of Tea Saponin on the Foaming Properties of Pea Protein. Food Funct..

[152] Shen Q., Zheng W., Han F., Zuo J., Dai J., Tang C., Song R., Li B., Chen Y. (2023). Air-Water Interfacial Properties and Quantitative Description of Pea Protein Isolate-Tween 20. Food Hydrocoll..

[153] Kapoor R., Karabulut G., Mundada V., Feng H. (2023). Non-Thermal Ultrasonic Contact Drying of Pea Protein Isolate Suspensions: Effects on Physicochemical and Functional Properties. Int. J. Biol. Macromol..

[154] Cui L., Bandillo N., Wang Y., Ohm J.-B., Chen B., Rao J. (2020). Functionality and Structure of Yellow Pea Protein Isolate as Affected by Cultivars and Extraction pH. Food Hydrocoll..

[155] McClements D.J., Lu J., Grossmann L. (2022). Proposed Methods for Testing and Comparing the Emulsifying Properties of Proteins from Animal, Plant, and Alternative Sources. Colloids Interfaces.

[156] Lam R.S.H., Nickerson M.T. (2013). Food Proteins: A Review on Their Emulsifying Properties Using a Structure–Function Approach. Food Chem..

[157] Liu Q., Dai Y., Hou H., Wang W., Ding X., Zhang H., Li X., Dong H. (2020). Changes in the Structure and Emulsification Properties of Pea Protein Isolate during Grinding. Food Sci. Technol..

[158] Peng W., Kong X., Chen Y., Zhang C., Yang Y., Hua Y. (2016). Effects of Heat Treatment on the Emulsifying Properties of Pea Proteins. Food Hydrocoll..

[159] Gao K., Zha F., Yang Z., Rao J., Chen B. (2022). Structure Characteristics and Functionality of Water-Soluble Fraction from High-Intensity Ultrasound Treated Pea Protein Isolate. Food Hydrocoll..

[160] Sha L., Koosis A.O., Wang Q., True A.D., Xiong Y.L. (2021). Interfacial Dilatational and Emulsifying Properties of Ultrasound-Treated Pea Protein. Food Chem..

[161] Wang W., Sun R., Ji S., Xia Q. (2024). Effects of κ-Carrageenan on the Emulsifying Ability and Encapsulation Properties of Pea Protein Isolate-Grape Seed Oil Emulsions. Food Chem..

[162] Lao Y., Ye Q., Wang Y., Vongsvivut J., Selomulya C. (2023). Quantifying the Effects of Pre-Roasting on Structural and Functional Properties of Yellow Pea Proteins. Food Res. Int..

[163] Cai W., Huang W., Chen L. (2021). Soluble Pea Protein Aggregates Form Strong Gels in the Presence of κ-Carrageenan. ACS Food Sci. Technol..

[164] Moreno H.M., Domínguez-Timón F., Díaz M.T., Pedrosa M.M., Borderías A.J., Tovar C.A. (2020). Evaluation of Gels Made with Different Commercial Pea Protein Isolate: Rheological, Structural and Functional Properties. Food Hydrocoll..

[165] Arntfield S.D., Maskus H.D. (2011). Peas and Other Legume Proteins. Handbook on Food Proteins.

[166] Barac M.B., Pesic M.B., Stanojevic S.P., Kostic A.Z., Bivolarevic V. (2015). Comparative Study of the Functional Properties of Three Legume Seed Isolates: Adzuki, Pea and Soy Bean. J. Food Sci. Technol..

[167] Xia S., Xue Y., Xue C., Jiang X., Li J. (2022). Structural and Rheological Properties of Meat Analogues from *Haematococcus pluvialis* Residue-Pea Protein by High Moisture Extrusion. LWT.

[168] Bu F., Feyzi S., Nayak G., Mao Q., Kondeti V.S.S.K., Bruggeman P., Chen C., Ismail B.P. (2023). Investigation of Novel Cold Atmospheric Plasma Sources and Their Impact on the Structural and Functional Characteristics of Pea Protein. Innov. Food Sci. Emerg. Technol..

[169] FAO (2013). Dietary Protein Quality Evaluation in Human Nutrition Report of an FAO Expert Consultation. Report of an FAO Expert Consultation.

[170] Guillin F.M., Gaudichon C., Guérin-Deremaux L., Lefranc-Millot C., Airinei G., Khodorova N., Benamouzig R., Pomport P.-H., Martin J., Calvez J. (2022). Real Ileal Amino Acid Digestibility of Pea Protein Compared to Casein in Healthy Humans: A Randomized Trial. Am. J. Clin. Nutr..

[171] Röhe I., Göbel T.W., Goodarzi Boroojeni F., Zentek J. (2017). Effect of Feeding Soybean Meal and Differently Processed Peas on the Gut Mucosal Immune System of Broilers. Poult. Sci..

[172] Rivera del Rio A., Boom R.M., Janssen A.E.M. (2022). Effect of Fractionation and Processing Conditions on the Digestibility of Plant Proteins as Food Ingredients. Foods.

[173] Ma Z., Boye J.I., Hu X. (2017). In Vitro Digestibility, Protein Composition and Techno-Functional Properties of Saskatchewan Grown Yellow Field Peas (*Pisum sativum* L.) as Affected by Processing. Food Res. Int..

[174] Çabuk B., Nosworthy M.G., Stone A.K., Korber D.R., Tanaka T., House J.D., Nickerson M.T. (2018). Effect of Fermentation on the Protein Digestibility and Levels of Non-Nutritive Compounds of Pea Protein Concentrate. Food Technol. Biotechnol..

[175] Skalickova S., Ridoskova A., Slama P., Skladanka J., Skarpa P., Smykalova I., Horacek J., Dostalova R., Horky P. (2022). Effect of Lactic Fermentation and Cooking on Nutrient and Mineral Digestibility of Peas. Front. Nutr..

[176] Compton M., Willis S., Rezaie B., Humes K. (2018). Food Processing Industry Energy and Water Consumption in the Pacific Northwest. Innov. Food Sci. Emerg. Technol..

[177] Yao S., Li W., Martin G.J.O., Ashokkumar M. (2023). An Investigation into the Mechanism of Alkaline Extraction-Isoelectric Point Precipitation (AE-IEP) of High-Thiol Plant Proteins. Appl. Sci..

[178] Du T., Xu J., Zhu S., Yao X., Guo J., Lv W. (2022). Effects of Spray Drying, Freeze Drying, and Vacuum Drying on Physicochemical and Nutritional Properties of Protein Peptide Powder from Salted Duck Egg White. Front. Nutr..

[179] Hou Y., Yang F., Cao J., Huang Y., Li C., Li J., Ren X. (2022). Effects of Hydrodynamic Cavitation at Different pH Values on the Physicochemical Properties and Aggregation Behavior of Soybean Glycinin. LWT.

[180] Gong Q., Liu C., Tian Y., Zheng Y., Wei L., Cheng T., Wang Z., Guo Z., Zhou L. (2023). Effect of Cavitation Jet Technology on Instant Solubility Characteristics of Soymilk Flour: Based on the Change of Protein Conformation in Soymilk. Ultrason. Sonochem..

[181] Donsì F., Ferrari G., Lenza E., Maresca P. (2009). Main Factors Regulating Microbial Inactivation by High-Pressure Homogenization: Operating Parameters and Scale of Operation. Chem. Eng. Sci..

[182] Martynenko A., Astatkie T., Satanina V. (2015). Novel Hydrothermodynamic Food Processing Technology. J. Food Eng..

[183] Panda D., Manickam S. (2019). Cavitation Technology-the Future of Greener Extraction Method: A Review on the Extraction of Natural Products and Process Intensification Mechanism and Perspectives. Appl. Sci..

[184] Boukid F. (2021). Plant-Based Meat Analogues: From Niche to Mainstream. Eur. Food Res. Technol..

[185] Ge J., Sun C., Corke H., Gul K., Gan R., Fang Y. (2020). The Health Benefits, Functional Properties, Modifications, and Applications of Pea (*Pisum sativum* L.) Protein: Current Status, Challenges, and Perspectives. Compr. Rev. Food Sci. Food Saf..

[186] Banki N.M., Salihu A., Muhammad A., Bala S.M. (2021). Optimization and Characterization of Rice–Pigeon Pea Flour Blend Using Extrusion Cooking Process. Legume Sci..

[187] Chan E., Masatcioglu T.M., Koksel F. (2019). Effects of Different Blowing Agents on Physical Properties of Extruded Puffed Snacks Made from Yellow Pea and Red Lentil Flours. J. Food Process Eng..

[188] Jebalia I., Maigret J.-E., Réguerre A.-L., Novales B., Guessasma S., Lourdin D., Della Valle G., Kristiawan M. (2019). Morphology and Mechanical Behaviour of Pea-Based Starch-Protein Composites Obtained by Extrusion. Carbohydr. Polym..

[189] Webb D., Plattner B.J., Donald E., Funk D., Plattner B.S., Alavi S. (2020). Role of Chickpea Flour in Texturization of Extruded Pea Protein. J. Food Sci..

[190] Saint-Eve A., Granda P., Legay G., Cuvelier G., Delarue J. (2019). Consumer Acceptance and Sensory Drivers of Liking for High Plant Protein Snacks. J. Sci. Food Agric..

[191] Morales-Polanco E., Campos-Vega R., Gaytán-Martínez M., Enriquez L.G., Loarca-Piña G. (2017). Functional and Textural Properties of a Dehulled Oat (*Avena sativa* L) and Pea (*Pisum sativum*) Protein Isolate Cracker. Food Sci. Technol..

[192] Kim T., Miller R., Laird H., Riaz M.N. (2021). Beef Flavor Vegetable Hamburger Patties with High Moisture Meat Analogs (HMMA) with Pulse Proteins—Peas, Lentils, and Faba Beans. Food Sci. Nutr..

[193] De Angelis D., Kaleda A., Pasqualone A., Vaikma H., Tamm M., Tammik M.-L., Squeo G., Summo C. (2020). Physicochemical and Sensorial Evaluation of Meat Analogues Produced from Dry-Fractionated Pea and Oat Proteins. Foods.

[194] Shin J.-S., Kim B.-H., Baik M.-Y. (2021). Applicable Plant Proteins and Dietary Fibers for Simulate Plant-Based Yogurts. Foods.

[195] Klost M., Drusch S. (2019). Structure Formation and Rheological Properties of Pea Protein-Based Gels. Food Hydrocoll..

[196] Yousseef M., Lafarge C., Valentin D., Lubbers S., Husson F. (2016). Fermentation of Cow Milk and/or Pea Milk Mixtures by Different Starter Cultures: Physico-Chemical and Sensorial Properties. LWT Food Sci. Technol..

[197] Akkam Y., Rababah T., Costa R., Almajwal A., Feng H., Laborde J.E.A., Abulmeaty M.M., Razak S. (2021). Pea Protein Nanoemulsion Effectively Stabilizes Vitamin d in Food Products: A Potential Supplementation during the COVID-19 Pandemic. Nanomaterials.

[198] Hadidi M., Motamedzadegan A., Jelyani A.Z., Khashadeh S. (2021). Nanoencapsulation of Hyssop Essential Oil in Chitosan-Pea Protein Isolate Nano-Complex. Food Sci. Technol..

